# Assessing the impact of long-term exposure to nine outdoor air pollutants on COVID-19 spatial spread and related mortality in 107 Italian provinces

**DOI:** 10.1038/s41598-022-17215-x

**Published:** 2022-08-03

**Authors:** Gaetano Perone

**Affiliations:** Department of Economics and Management, University of Pisa, Via Cosimo Ridolfi n. 10, 56124 Pisa, Italy

**Keywords:** Environmental sciences, Diseases, Health care, Risk factors

## Abstract

This paper investigates the air quality in 107 Italian provinces in the period 2014–2019 and the association between exposure to nine outdoor air pollutants and the COVID-19 spread and related mortality in the same areas. The methods used were negative binomial (NB) regression, ordinary least squares (OLS) model, and spatial autoregressive (SAR) model. The results showed that (i) common air pollutants—nitrogen dioxide (NO_2_), ozone (O_3_), and particulate matter (PM_2.5_ and PM_10_)—were highly and positively correlated with large firms, energy and gas consumption, public transports, and livestock sector; (ii) long-term exposure to NO_2_, PM_2.5_, PM_10_, benzene, benzo[a]pyrene (BaP), and cadmium (Cd) was positively and significantly correlated with the spread of COVID-19; and (iii) long-term exposure to NO_2,_ O_3_, PM_2.5_, PM_10_, and arsenic (As) was positively and significantly correlated with COVID-19 related mortality. Specifically, particulate matter and Cd showed the most adverse effect on COVID-19 prevalence; while particulate matter and As showed the largest dangerous impact on excess mortality rate. The results were confirmed even after controlling for eighteen covariates and spatial effects. This outcome seems of interest because benzene, BaP, and heavy metals (As and Cd) have not been considered at all in recent literature. It also suggests the need for a national strategy to drive down air pollutant concentrations to cope better with potential future pandemics.

## Introduction

The coronavirus disease of 2019 (COVID-19) is a severe acute respiratory syndrome that officially appeared for the first time in Wuhan, a city in the Hubei province of China, in December 2019. From the end of February 2020, the virus was rapidly spreading across the globe, dramatically changing every aspect of people’s lives. As of 1 November 2021, the COVID-19 pandemic had affected almost all countries in the world, with about 250 million confirmed cases and more than 5 million deaths^1^. At the time of writing, the virus has been mutating by generating new forms or variants of itself—the most important of which were first found in the UK, South Africa, Brazil, and India^2^—making the fight against the outbreak even more difficult. In fact, many countries which are approaching the third or even fourth wave of infections have had to reintroduce or extend their lockdowns and social distancing measures. The worst-hit countries include both advanced and developing ones, such as Brazil, France, India, Italy, Russia, Turkey, the UK, and the US.

In these circumstances, it has become crucial to identify the optimal containment and mitigation policies to prevent and manage the spread of the outbreak and prepare a plan to tackle the risk of future epidemics and pandemics. In the last year, a closer look has been taken at the potential adverse impact of air pollution on the spread dynamic and death toll of COVID-19. In fact, it is widely recognized that several air pollutants, such as benzo[a]pyrene (BaP), nitrogen dioxide (NO_2_), ozone (O_3_), particulate matter (PM), and sulfur dioxide (SO_2_), can cause irritation, inflammation, and serious infections and diseases to the lungs and airways^3–5^. This is a matter of great concern, considering that according to an EEA report [Ref.^6^, pp. 40, 42], the annual emissions of PM_2.5_ and PM_10_ in 2018 exceeded the limits set by the World Health Organization^7^ Air Quality Consultant (AQG) at 70% and 53% of the stations spread across European countries, respectively.

In particular, the relationship between air pollution exposure and COVID-19 revealed that poor air quality may have favored COVID-19 transmissibility around the world^8–13^ and may have enhanced the risk of severe and fatal COVID-19^14–19^.

This study may be of interest for two main reasons. First, as of 1 November 2021, Italy is one of the most affected countries worldwide, with 4,796,929 confirmed cases, that is, about 8% of the whole resident population, and 132,263 confirmed deaths. Second, although the literature has already established a positive and significant relationship between air pollution and COVID-19 spread/mortality in Italy^9,12,19–26^, these studies may have suffered from some limitations: (i) they mainly focused on a number of regions and provinces and referred to the early phase of the outbreak; (ii) in many cases, they focused on the impact of short-term exposure to common air pollutants—NO_2_, O_3_, PM, and SO_2_—on COVID-19 infections and deaths; iii) they did not consider other potentially dangerous air pollutants, such as polycyclic aromatic hydrocarbons (PAHs) and heavy metals; (iv) they did not consider other important covariates (except for Refs.^12,19^), such as demographic characteristics, weather conditions, population habits and structure, and industrial centers; and (v) finally, they did not explicitly consider the spatial dependency of COVID-19 infections, that is, the possibility that neighboring territories may have affected each other through the movement of people.

In this study, I try to fill this gap by jointly considering all these aspects. Thus, the goals of this study are the following: (i) I investigate the general air quality in the Italian provinces in the period 2014–2019, trying to assess the main sources of outdoor air pollution and identifying the most polluted territories in the country; and (ii) I use negative binomial (NB) regression model, an ordinary least squares (OLS) econometric approach, and spatial autoregressive (SAR) model to assess the relationship between long-term exposure to nine air pollutants in the period 2014–2019—NO_2_, O_3_, PM_2.5_, PM_10_, benzene, BaP, arsenic (As), cadmium (Cd), and nickel (Ni)—and COVID-19 spread and related mortality at the second peak of the outbreak. [Note 1: This is an important task because the risk of multiple COVID-19 waves is real^27–29^.

The rest of the paper is organized as follows. “Environmental pollution in the Italian provinces” discusses the air quality in the Italian provinces; “Literature review” discusses the related literature; “Data” presents the data used in the empirical analysis; “Empirical strategy” discusses the empirical strategy; “Results and discussion” presents and discusses the results; "Limitations" discusses the main study limitations; and finally, “Conclusions” provides some conclusive considerations.

## Environmental pollution in the Italian provinces

In this section, the main sources of nine air pollutants and the general quality of air in the 107 Italian provinces are investigated. According to European Environment Agency^30^, industry processes, road transport, agricultural activities, waste management, energy production and distribution (especially from fossil sources), natural phenomena (i.e., volcanic eruptions, sandstorms, etc.), public buildings, and households are the main causes of outdoor air pollution. For instance, exhaust emissions from vehicles and the abrasion of pneumatics and brakes can release benzene_,_ Cd, carbon monoxide (CO_2_), lead (Pb), mercury (Me), NO_2_, PM_2.5_, PM_10_, and sulfur oxides (SO_x_) into the atmosphere^31,32^ and favor chemical reactions that increase the likelihood of O_3_ formation. Business activities, livestock buildings, and households are the major factors responsible for production of PM_2.5_^33^. Industrial activities burning fuels (coal, petroleum, wood, etc.), components of smoke cigarettes, forest fires, and vehicle exhaust emissions are the main causes of benzene and BaP^34,35^.

Thus, in Table 1, I report the Spearman’s rank correlation coefficient between the nine investigated air pollutants (described in Table B1, Appendix B) and six potential sources of environmental pollution in the period 2014–2019: big firms with over 250 employees per square kilometer in the period 2014–2019; final consumption of energy and natural gas expressed as tons of oil equivalent per square kilometer in the period 2014–2019; number of vehicles used to transport goods and passengers (cars, motorcycles, and other vehicles) per square kilometer in the period 2014–2019; overall supply of local public transport expressed as number of seats per inhabitants in the period 2014–2019; the production of cattle fodder from permanent grassland expressed as quintal per square kilometer in the period 2014–2019; and the number of livestock (bovines, buffalos, and pigs) per square kilometer in the period 2014–2019. [Note 2: Data on energy and gas consumption, vehicles density, and public transports refer to the provincial capital; while data on big firms and production of cattle fodder are at provincial level. Only data on livestock density are at regional level]. Data were extracted from I.Stat database^36^, except for energy and gas consumption^37,38^, supply of local public transport^39^, and number of vehicles used to transport goods and passengers^38,40^. The results show that common air pollutants are positively and significantly correlated with big firms, energy and gas consumption, density of vehicles, public transport, cattle fodder, and livestock density. Big firms, energy and gas consumption, and livestock density had the highest rank correlation coefficients. Notably, NO_2_, O_3 (>120)_, O_3 (>180)_, PM_2.5_, and PM_10 (>50)_ showed rank correlation coefficients ranging from 0.58 to 0.68 for large firms, from 0.51 to 0.72 for energy and gas consumption, and from 0.38 to 0.71 for livestock density. This may have been partially caused by the ammonia (NH_3_) generated in the urine and feces of cattle^41,42^, which contributes to the formation of two relevant (secondary) components of particulate matter, ammonium nitrate and ammonium sulphate^43^. In fact, according to Greenpeace and the Italian Institute for Environmental Protection and Research (ISPRA)^44^, animal husbandry was the second leading cause of air pollution in Italy in the period 1990–2018, accounting for 17% of all PM_2.5_ formation.

**Table 1:** Spearman’s rank correlation coefficients between nine air pollutants and six potential sources of environmental pollution.

Air pollutants	Large firms per km^2^	Energy and gas consumption per km^2^	Vehicles per km^2^	Public transport	Cattle fodder per km^2^	Livestock per km^2^
**Common air pollutants**						
NO_2_	0.6754***	0.7167***	0.625***	0.4171***	0.3244***	0.3762***
O_3 (>120)_	0.5833***	0.5494***	0.3064***	0.268***	0.5395***	0.6381***
O_3 (>180)_	0.6322***	0.5834***	0.3729***	0.3675***	0.5073***	0.7044***
PM_2.5_	0.6126***	0.5831***	0.385***	0.2672***	0.446***	0.6897***
PM_10_	0.5838***	0.5117***	0.42***	0.2179**	0.3963***	0.5688***
PM_10 (>50)_	0.5957***	0.5639***	0.4139***	0.2177**	0.4899***	0.708***
**PAHs**						
Benzene	0.3925***	0.3989***	0.4618***	0.272**	0.0829	0.1291
BaP	0.2047*	0.3689***	0.2026*	0.0904	0.359***	0.2456**
**Heavy metals**						
As	0.2726**	0.2627**	0.1493	− 0.0204	0.2322*	0.3931***
Cd	0.341***	0.3432**	0.2723**	0.2307*	0.0931	0.0975
Ni	0.1285	0.2702**	0.2439*	0.131	0.1609	− 0.0641

p-value < 0.01***; p-value < 0.05**; p-value < 0.1*.

Among PAHs, benzene is positively correlated with big firms, energy and gas consumption, and vehicle density at 1% level of significance, and with public transport at 5% level of significance. BaP is positively associated with energy and gas consumption and cattle fodder production at 1% level of significance, and with livestock density at 5% level of significance. Heavy metals are significantly and positively correlated especially with large firms and energy and gas consumption. Notably, the Spearman’s rank correlation coefficients for PAHs and heavy metals are lower than those for common air pollutants. Although these correlations do not imply causation, they warn of the potentially dangerous effects of large firms, vehicles, energy and gas consumption, and livestock sector.

This is particularly worrying because according to the Air Quality Standards established by the European Commission^45^, the legal threshold for key air pollutants was violated multiple times by most of the Italian provinces in the period 2014–2019 (Table 2). Specifically, almost all provinces (106 out of 107) violated the PM_10_ limit of 50 µg/m^3^ both in the short- and long-term, resulting in a national average of 25.15 violations per year. Notably, the legal thresholds for both measures of O_3_ were also violated several times both in the short- and long-term, with a maximum of 95 provinces involved. Regarding the average concentrations of NO_2_, PM_2.5_, and PM_10_, the violations were fewer, respectively involving 15, 17, and five provinces in the short-term and 11, four, and no provinces in the long-term. Among the PAHs, the legal limit for BaP was violated by 13 provinces in the short-term and seven provinces in the long-term, while the legal threshold for benzene was never exceeded. No provinces registered violations for heavy metals, except Aosta and Terni, which exceeded the legal limit of Ni in the short-term.

**Table 2:** Provinces that exceeded the EU legal threshold in the period 2014–2019.

Air pollutants	EU legal threshold	National averages	Provinces with long-term violations	Provinces with at least 1-year violation
NO_2_	40 µg/m^3^	26.3279	11	15
O_3_	> 120 µg/m^3^	28.2256	95	95
O_3_	> 180 µg/m^3^	8.9064	58	58
PM_2.5_	25 µg/m^3^	15.3609	4	17
PM_10_	40 µg/m^3^	24.952	0	5
PM_10_	> 50 µg/m^3^	25.1509	106	106
Benzene	5 µg/m^3^	1.2069	0	0
BaP	1 ng/m^3^	0.4363	7	13
As	6 ng/m^3^	0.9559	0	0
Cd	5 ng/m^3^	0.343	0	0
Ni	20 ng/m^3^	3.6301	0	2

Source: European Commission^45^.

The situation becomes even worse when we consider the most restrictive legal thresholds set by the World Health Organization^46^. In this case, the legal threshold for PM_2.5_ and PM_10_ was violated respectively by 88 and 93 provinces in the short-term and by 85 and 86 provinces in the long-term (Table 3). Unlike EU law, the WHO has not established safe limits for the PAHs (benzene and BaP) and heavy metals (As and Ni) considered, except for Cd, which remains unchanged. This is not very surprising because according to the EEA^47^, Italian and Polish cities were the ones with the highest levels of PM_2.5_ in the period 2019–2020, among 323 investigated localities. In fact, among Europe’s 53 worst cities for PM_2.5_ levels, 20 were in Italy.

**Table 3:** Provinces that exceeded the WHO AQG threshold in the period 2014–2019.

Air pollutants	WHO AQG threshold	National averages	Provinces with long-term violations	Province with at least 1-year violation
NO_2_	40 µg/m^3^	26.3279	11	15
O_3 (8 h)_	> 100 µg/m^3^	28.2256^a^	95^a^	95^a^
PM_2.5_	10 µg/m^3^	15.3609	85	88
PM_10_	20 µg/m^3^	24.952	86	93
PM_10_	> 50 µg/m^3^	25.1509	106	106
Benzene	No safe level	1.2069	–	–
BaP	No safe level	0.4363	–	–
As	No safe level	0.9559	–	–
Cd	5 ng/m^3^	0.343	0	0
Ni	No safe level	3.6301	–	–

Source: WHO^46^.

^a^Due to a lack of data, these violations referred to the legal threshold limit of 120 µg/m^3^.

In Table 4, I also calculate a synthetic environmental pollution index for the Italian provinces in the period 2014–2019, using data on NO_2_, O_3 (>120)_, PM_2.5_, and PM_10_, for which there are sufficient observations. Specifically, the index is compiled by switching the data on each of the four air pollutants considered to fixed-base indexes (with average = 1), from whose arithmetic mean I achieve the final standardized index. Provinces are ranked from the most polluted to the cleanest.

**Table 4:** A synthetic environmental pollution index for the Italian provinces in the period 2014–2019.

Province	Index	Province	Index
**1-Monza and Brianza**	**1.7639**	55-Ascoli Piceno	0.8696
**2-Brescia**	**1.6969**	56-Rieti	0.8675
**3-Milan**	**1.6681**	57-Avellino	0.8572
**4-Bergamo**	**1.6278**	58-Caserta	0.8464
**5-Lodi**	**1.6175**	59-Bari	0.8408
**6-Cremona**	**1.6097**	60-Foggia	0.8315
**7-Turin**	**1.5723**	61-Livorno	0.8237
**8-Pavia**	**1.5631**	62-Pescara	0.8195
**9-Mantua**	**1.5302**	63-Perugia	0.8118
**10-Alessandria**	**1.5261**	64-Syracuse	0.8086
11-Piacenza	1.5229	65-Aosta	0.8058
12-Vicenza	1.512	66-Isernia	0.8012
13-Como	1.4881	67-Crotone	0.7981
14-Varese	1.4826	68-Teramo	0.7977
15-Genoa	1.4759	69-Pisa	0.7955
16-Venice	1.4727	70-Ancona	0.7936
17-Padua	1.451	71-Grosseto	0.7822
18-Modena	1.4293	72-Campobasso	0.7798
19-Verona	1.4267	73-Massa-Carrara	0.7749
20-Parma	1.4176	74-Benevento	0.7702
21-Treviso	1.3975	75-La Spezia	0.7599
22-Lecco	1.3836	76-Cosenza	0.7556
23-Reggio Emilia	1.3724	77-Siena	0.7352
24-Vercelli	1.30156	78-Cagliari	0.7232
25-Rovigo	1.2989	79-Latina	0.7228
26-Rimini	1.2815	80-Taranto	0.7076
27-Novara	1.2781	81-Savona	0.6869
28-Bologna	1.2741	82-Brindisi	0.6868
29-Ferrara	1.2291	83-Vibo Valentia	0.6826
30-Naples	1.2182	84-L’Aquila	0.6815
31-Frosinone	1.2172	85-Enna	0.6806
32-Trento	1.2082	86-Imperia	0.6743
33-Florence	1.2056	87-Salerno	0.6518
34-Terni	1.1402	88-Macerata	0.6335
35-Prato	1.0913	89-Barletta-Andria-Trani	0.6168
36-Forlì-Cesena	1.0686	90-Viterbo	0.6166
37-Pordenone	1.0526	91-Catania	0.6118
38-Asti	1.0452	92-Lecce	0.574
39-Udine	1.0426	93-Pistoia	0.5671
40-Ravenna	1.0379	94-Potenza	0.5539
41-Chieti	1.0297	95-Reggio Calabria	0.5467
42-Cuneo	1.024	96-Ragusa	0.5457
43-Sondrio	0.988	97-Catanzaro	0.5352
44-Palermo	0.9861	*98-Oristano*	*0.5269*
45-Gorizia	0.9729	*99-Caltanissetta*	*0.5237*
46-Rome	0.9632	*101-Messina*	*0.5131*
47-Biella	0.9541	*102-Agrigento*	*0.5065*
48-Lucca	0.9168	*103-Fermo*	*0.4961*
49-Verbano-Cusio-Ossola	0.91	*104-Sassari*	*0.496*
50-Arezzo	0.9034	*105-South Sardinia*	*0.4654*
51-Pesaro and Urbino	0.892	*105-Trapani*	*0.3985*
52-Trieste	0.8747	*106-Matera*	*0.3825*
53-Belluno	0.8729	*107-Nuoro*	*0.3741*
54-Bolzano	0.8729		

The provinces are ranked from the most polluted to the cleanest. The 10 most polluted provinces are bold, while the 10 cleanest provinces are italics.

The output shows that the top positions are all in Northern Italy. In particular, the 29 most polluted Italian provinces are all concentrated in the eight northern regions of Italy. Among them, the top six positions are held by provinces within Lombardy, that is, the Italian region which has been most severely hit by the COVID-19 outbreak.

On the contrary, the southern provinces hold the lowest positions in the ranking. In the bottom 20 positions of the ranking, 16 are southern provinces, only four provinces are in Central Italy (Fermo, Macerata, Pistoia, and Viterbo), and none are in Northern Italy.

While the most polluted southern provinces are Naples and Chieti, they are in 29th and 41st place, respectively. The results reflect the deep historical gap in industrialization and development between the north and south of Italy^48,49^.

**Figure 1:**
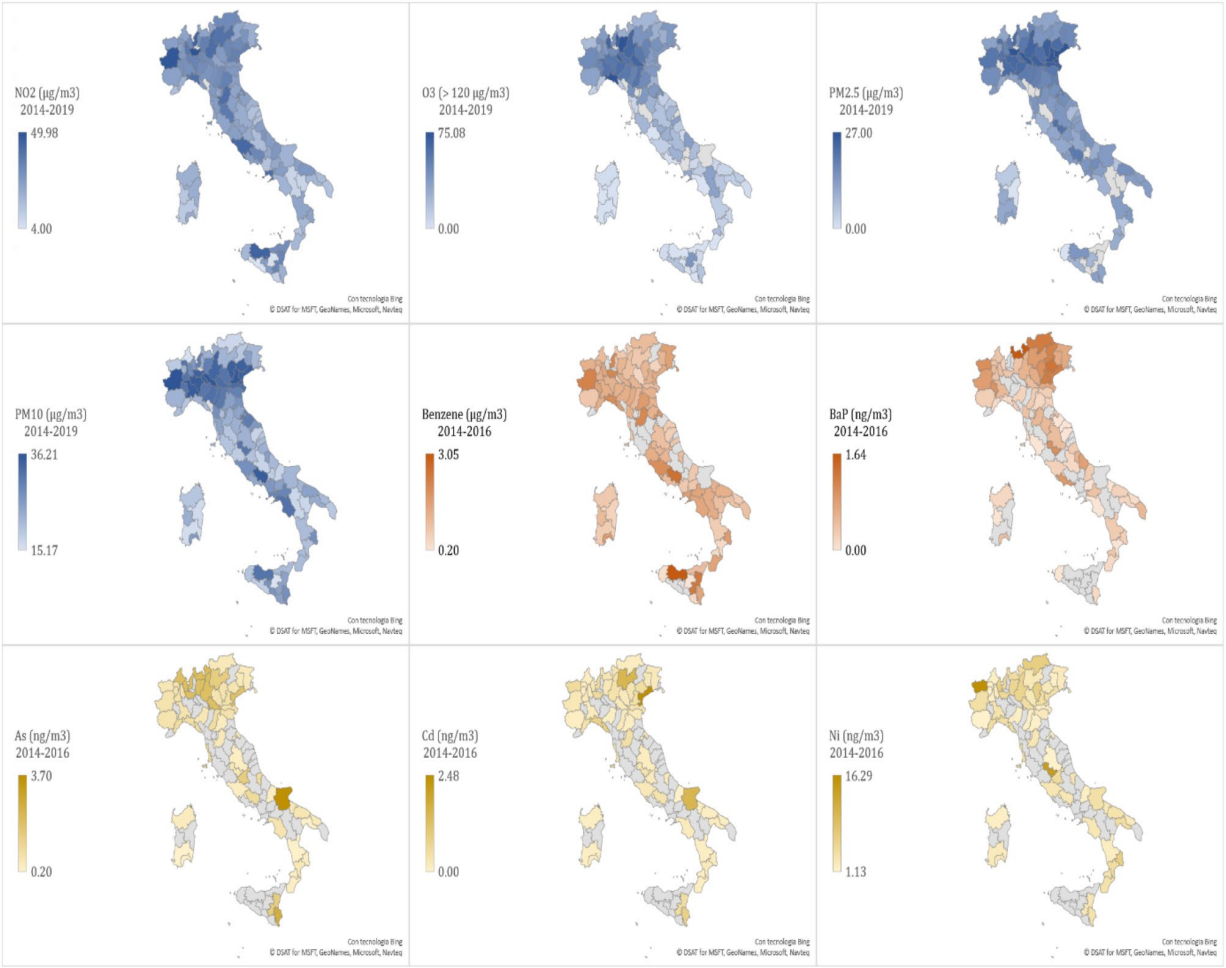
Average long-term outdoor concentrations (or violations) of NO_2_, O_3_, PM_2.5_, PM_10_, benzene, BaP, As, Cd, and Ni, in the 107 Italian provinces. When no data are available, the province is grey colored. The map was generated using Microsoft Excel software 2021. All the sources used to collect the data are reported in detail in the Appendix B.

An air pollution map for the average long-term concentrations or violations of each air pollutant in the Italian provinces is given in Fig. 1.

## Literature review

It is well-established that air pollution exposure can adversely affect lung function. NO_2_, O_3_, PM_2.5_, and PM_10_ can be risk factors for several respiratory diseases, such as asthma^50^, bronchiectasis^51^, chronic obstructive pulmonary disease (COPD)^52^, invasive pneumococcal disease (IPD)^53^, lung cancer^54^, and general respiratory infections^55^. Meanwhile, exposure to airborne PAHs can worsen respiratory infections and increase the risk of several non-malignant respiratory diseases associated with exposure to other air pollution, such as particulate matter^56^. Exposure to heavy metals, such as As, Cd, chromium (Cr), mercury (Hg), Ni, and Zinc (Zn), may induce airway inflammation, lung irritation, and pulmonary oedema^57–60^, contributing to oxidative stress in lung tissue^61–63^. E.g., Cd and Ni exposure may lead to emphysema and asthma, respectively^57^, while arsenic exposure may increase the risk of developing pulmonary fibrosis^64^.

Therefore, in the last year and a half, a large body of literature has focused its attention on the relationship between air quality and the COVID-19 pandemic propagation pattern and mortality. Bashir et al.^8^ used two non-parametric statistical techniques—Kendal and Spearman rank-order correlation coefficients—to investigate the association between seven air pollutants and COVID-19 cases and deaths in California. Specifically, they analyzed the concentrations of CO, NO_2_, Pb, PM_2.5_, PM_10_, SO_2_, and volatile organic compounds (VOC) from 4 March 2020 to 24 April 2020. They found that short-term exposure to CO, NO_2_, PM_2.5_, PM_10_, and SO_2_ was significantly and positively correlated with COVID-19 cases and deaths, and the highest correlation coefficients were shown by NO_2_ and PM_2.5_.

Becchetti et al.^19^ used several statistical techniques, such as the difference-in-difference (DID) approach, ordinary least square (OLS) panel regression, and cross-sectional and panel fixed-effect spatial autoregressive combined models (SAC), to investigate the role of three major air pollutants in the spread of COVID-19 in 96 Italian provinces from 24 February 2020 to 15 April 2020. They found that average concentrations of NO_2_, PM_2.5_, and PM_10_ (registered in 2018) were highly significant and positively associated both with COVID-19 mortality and infections. The results were also confirmed after controlling for a number of demographic, environmental, economic, and healthcare covariates.

By using a mixed linear multiple regression approach, Hendryx and Luo^15^ analyzed the effect of long-term exposure (in the period 2014–2019) to diesel particulate matter (DPM), O_3_, and PM_2.5_ in relation to COVID-19 susceptibility or outcomes in the US. Specifically, they investigated the cumulative confirmed cases as of 31 May 2020, finding that DPM alone was significantly and positively associated with COVID-19 prevalence, and robust enough against changes in the specifications. Although positive, the coefficient of PM_2.5_ was not robust enough.

Cole et al.^14^ examined the link between confirmed COVID-19 cases, deaths, hospitalizations, and long-term exposure (in the period 2010–2019) to three major air pollutants (O_3_, PM_2.5_, and SO_2_) in 355 municipalities in The Netherlands. By using instrumental variable (IV) regressions, NB approaches, and spatial autoregressive models with autoregressive disturbances (SARAR), they found that only the PM_2.5_ coefficient was significant and robust against changes in the specifications. Specifically, for every 1 µg/m^3^ increase in PM_2.5_ concentrations, there was an increase of 9.4 cases, 2.3 deaths, and three hospitalizations.

Liang et al.^65^ used zero-inflated negative binomial (ZINB) models to analyze the association between long-term exposure (in the period 2010–2016) to NO_2_, O_3_, and PM_2.5_, and COVID-19 case-fatality and mortality rates in 3,076 US counties. They found that only NO_2_ had a significant and positive association with both COVID-19 case-fatality rate and mortality rate from 22 January 2020 to 17 July 2020.

By using a generalized additive model (GAM), Zhu et al.^11^ investigated the short-term relationship between several air pollutants and daily confirmed COVID-19 cases in 120 Chinese cities from 23 January 2020 to 29 February 2020. They found that 1-unit µg/m^3^ increases in NO_2_, O_3_, PM_2.5_, and PM_10_ were associated with 0.69%, 0.48%, 0.22%, and 0.18% increases respectively in daily confirmed COVID-19 cases. On the contrary, a 1-unit µg/m^3^ increase in SO_2_ was linked with a 0.78% decrease in daily confirmed COVID-19 cases.

Dales et al.^66^ analyzed the relationship between short-term exposure to CO, NO_2_, and PM_2.5_ and COVID-19 related mortality in Santiago (Chile). They used a two-stage random effects model for count data in the period 16 March 2020–31 August 2020. In particular, they found that daily deaths from COVID-19 related mortality grew by 6% for an interquartile range (IQR) increase in CO, NO_2_, and PM_2.5_. No significant effects were detected for O_3_.

Solimini et al.^13^ used negative binomial mixed–effect models (NBMM) to investigate the association between long-term exposure (in the period 2015–2018) to PM_10_ and PM_2.5_ and COVID-19 cases in a large sample of countries. The data came from 63 countries, 730 regions, and five continents, and was updated on 30 May 2020. After adjusting the models for several regional and country covariates and spatial correlation, they found that 1-unit µg/m^3^ increases in the PM_2.5_ and PM_10_ concentrations were significantly correlated with increases of 0.81% and 1.15% respectively in the total number of confirmed COVID-19 cases in a 14-day window.

Table 5 summarizes 25 international studies on the relationship between environmental pollution and the spread of COVID-19 infections.

**Table 5:** 25 Selected studies on the relationship between exposure to air pollution and COVID-19 spread and related mortality across the world.

Author	Area	Method	COVID-19 cases	COVID-19 deaths
^8^	California	Kendall and Spearman correlation	CO (+), NO_2_ (+), PM_2.5,_ (+), PM_10_ (+), SO_2_ (+)	N/a
^67^	10 big cities from Latin America and the Caribbean	Spearman correlation	NO_2_, PM_2.5,_ and PM_10_ (+) in São Paulo, Santiago, San Juan, and Buenos Aires, and (−) in Bogotá and Mexico City	NO_2 ,_ PM_2.5,_, and PM_10_ (+) in São Paulo, Santiago, and Buenos Aires, and (−) in Mexico City
^21^	55 Italian provinces	OLS, quadratic model	O_3_ (+), PM_10_ (+)	N/a
^14^	355 Dutch municipalities	IV, NB, SARAR	PM_2.5_ (+), NO_2_ (+)	PM_2.5_ (+), NO_2_ (+)
^68^	Modena and Ravenna (Italy)	Granger causality	PM_2.5_ (+), PM_10_ (+)	N/a
^23^	71 Italian provinces	Pearson correlation	NO_2_ (+), O_3_ (+), PM_2.5_ (+), PM_10_ (+)	N/a
^9^	28 Italian provinces	Multivariable RCS regression	NO_2_ (+)	N/a
^15^	3143 US counties	Mixed linear multiple regression	DPM (+), PM_2.5_ (+)	DPM (+), PM_2.5_ (+)
^69^	23 Viennese districts (Austria)	Cox regression	NO_2_ (+), PM_10_ (+)	NO_2_ (+)
^65^	3,076 US counties	ZINB	N/a	NO_2_ ( +)
^70^	Wuhan and XiaoGan (China)	Pearson correlation	AQI (+), PM_2.5_ (+), and NO_2_ (+)	N/a
^71^	29 China provinces	Pearson/Spearman correlation	CO (+), NO_2_ (−)	N/a
^24^	Florence, Milan, Trento (Italy)	Spearman/Kendall correlation	PM_2.5_ (+)	N/a
^16^	World	EMAC	N/a	PM_2.5_ (+)
^72^	24 Districts of Metropolitan Lima (Peru)	Pearson correlation	PM_2.5_ (+)	PM_2.5_ (+)
^11^	120 Chinese cities	GAM	NO_2_ (+), O_3_ (+), PM_2.5_ (+), PM_10_ (+), SO_2_ (−)	N/a
^10^	2,019 Chinese cities	Spearman/Kendall correlation, OLS	AQI (+)	N/a
^25^	Milan (Italy)	Pearson correlation	AQI (+), PM_2.5_ (+), PM_10_ (+)	N/a
^66^	Santiago (Chile)	Two-stage random effects	N/a	CO (+), NO_2_ (+), PM_2.5_ (+)
^12^	1439 municipalities of Lombardy (Italy)	NBMM	NO_2_ (−), PM_2.5_ (+), PM_10_ (+),	NO_2_ (−), PM_2.5_ (+)
^73^	Mexico City	Probit regression	N/a	PM_2.5_ (+)
^17^	Italian regions (20) and provinces (107)	OLS	N/a	NO_2_ (+), O_3_ (+), PM_2.5_ (+), PM_10_ (+)
^13^	730 regions (in 63 countries)	NBMM	PM_2.5_ (+), PM_10_ (+)	N/a
^18^	England (regional, sub-regional and individual data)	NB	Regional: NO_x_ (−), NO_2_ (+) Sub-regional: NO_x_ (+), NO_2_ (+), O_3_ (−) PM_2.5_ (−), PM_10_ (−) Individual: NO_x_ (+), NO_2_ (+), PM_2.5_ (+), PM_10_ (+)	Regional: NO_x_ (−), NO_2_ (+) O_3_ (+) Sub-regional: NO_x_ (+), NO_2_ (+), O_3_ (−)
^19^	96 Italian provinces	DID, OLS, SAC	NO_2_ (+), PM_2.5,_ (+), PM_10_ (+)	NO_2_ (+), PM_2.5,_ (+), PM_10_ (+)

*AIQ* air quality index, *DID* difference-in-difference, *EMAC* global atmospheric chemistry general circulation, *FE* fixed effect, *GAM* generalized additive model, *IV* instrumental variables, *N/a* not available, *NB* negative binomial, *NBMM* negative binomial mixed–effect model, *OLS* ordinary least square, *RCS* restricted cubic spline, *SAC* spatial autoregressive combined models, *ZINB* zero-inflated negative binomial. Only significant associations are reported.

## Data

In this section, I report the variables used in the empirical analysis. First, to avoid spurious correlations and mitigate the problem of omitted variables, I implement 18 covariates to account for geographical proximity, demographic characteristics, population habits and structure, industrial centers, and weather conditions:

- four dummy variables to identify the provinces that border Austria, France, Slovenia, and Switzerland respectively;
- a dummy variable to identify the provinces that are also the regional capital;
- the size of each province expressed in square kilometers;
- the distance between the provincial capital’s center and the nearest airport with at least 50,000 passengers in the period from January to November 2020;
- the foreign-born population as a percentage of total resident population in each province, in 2020;
- the share of population aged 0–19 in each province, in 2020;
- the share of male population in each province, in 2020;
- the degree of urbanization of the population in each province;
- the average share of obese individuals at regional level, in the period 2016–2019;
- the average share of smokers at regional level, in the period 2016–2019;
- the average deaths from chronic lower respiratory tract disease (per 100,000 inhabitants) in each province, in the period 2014–2019;
- the number of firms with 250 or more employees per 100 square kilometers in each province, in the period 2014–2019;
- the average altitude of the capital of the province;
- the average annual days of rain in each province, in the period 2007–2018;
- the average annual temperature in each province, in the period 2008–2018.

[Note 3: In Table A1 (Appendix A), I considered the pairwise correlation between the main control variables. The reported correlation coefficients for each pair of variables were always lower than the typical cutoff of 0.80^74^, and only 4 (out of 72) correlations were greater than the most restrictive cutoff of 0.5^75^, ranging from 0.51 to 0.61 (in absolute value). In Table A2 (Appendix A), I considered the pairwise correlation between control variables and air pollutants. Only 6 (out of 117) correlations were greater than the restrictive cutoff of 0.5^75^, ranging from 0.55 to 0.62 (in absolute value). This allows to strongly advocate the simultaneous inclusion of the covariates and single air pollutants].

What follow is a brief literature summary of the relationship between the main control variables and the spread and mortality of COVID-19. First, sex and age composition of population may be an important parameter in explaining the current outbreak. Some studies found that male population was more susceptible to contract COVID-19 infection^76^, and to have fatal outcomes than female population^77,78^. Young people and children are less likely to have severe and mild symptoms of COVID-19—such as fever and respiratory symptoms—than adults. Since they usually escape detection by health surveillance system, they could act as silent vectors of COVID-19 transmission^79,80^.

Population distribution may also affect transmission patterns of COVID-19 because in most densely populated and urban areas the spatial proximity means that people are more likely to contact other individuals^81^. This may contribute to spreading the contagion and exacerbate COVID-19 related mortality, such as observed in Brazil^82^, India^83^, and Italy^84^.

A number of studies found that the presence of at least one comorbidity, such as chronic lung disease and obesity, may have an adverse impact on patients with COVID-19^85,86^. In particular, comorbid respiratory and lung disease were found to be associated with higher COVID-19 prevalence^87^, and with higher risk for severe disease and mortality in COVID-19^87,88^. Similarly, higher prevalence of obesity may have increased the risk of severe COVID-19 outcomes for hospitalized patients in Milan, Italy^89^, in the UK^90^ and in New York City, US^91^.

The smoking habit in the population may have also played a role in the spread of COVID-19. In fact, even if the relationship between smoking and COVID-19 disease remain substantially unclear^92,93^, several studies found a partially unexpected protective effect of smoking/nicotine against COVID-19^94–96^. Active smokers were less likely to be infected with COVID-19 than non-smokers, by suggesting the existence of a smokers’ paradox in COVID-19^96^.

Predictive meteorological and geographical factors were also widely investigated. A number of studies found that higher altitude can mitigate the adverse effect of COVID-19 (transmission and related deaths) in Colombia^97^, Peru^98^, and the US^99^. Huamaní et al.^100^ suggested that, in Peruvian districts, this may be caused by the combination of low population density and smaller population. The results of the impact of average temperature on COVID-19 cases were mixed. If some studies found that temperature may increase the spread of COVID-19^8,101^, other research found a negative statistical association between the two variables^102,103^.While, there is a substantial consensus in the literature that warmer climate conditions may reduce COVID-19 mortality^103–105^ and case-fatality rate^17^. Even if rainfall was not found to be an important predictive factor in COVID-19 spread and mortality in the most literature^106^, a recent paper pointed out that rainfall may lead to higher social distancing and help to mitigate the adverse effects of the outbreak^107^. The provinces with international borders, the share of foreign population, the province capital’s distance from the nearest airport, and the size of the province are used to control the effect of the movement of people. Finally, the large firms can be seen as a proxy for greenhouse gases, such as CO_2_ and methane.

Regarding the explanatory variables, I chose the following nine air pollutants, calculated—when data are available—for each Italian province:

- the average concentrations of NO_2_, expressed in micrograms per cubic meter of air (µg/m^3^), in the period 2014–2019;
- the average number of days in which Ozone exceeded the limit of 120 µg/m^3^, in the period 2014–2019;
- the average number of hours in which Ozone exceeded the limit of 180 µg/m^3^, in the period 2014–2018;
- the average concentrations of PM_2.5_, expressed in µg/m^3^, in the period 2014–2019;
- the average concentrations of PM_10_, expressed in µg/m^3^, in the period 2014–2019;
- average number of days in which PM_10_ exceeded the limit of 50 µg/m^3^ in the period 2014–2018;
- the average concentrations of benzene, expressed in µg/m^3^, in the period 2014–2016;
- the average concentrations of BaP, expressed in nanogram per cubic meter of air (ng/m^3^), in the period 2014–2018;
- the average concentrations of As, expressed in ng/m^3^, in the period 2014–2016;
- the average concentrations of Cd, expressed in ng/m^3^, in the period 2014–2016;
- the average concentrations of Ni, expressed in ng/m^3^, in the period 2014–2016.

**Figure 2:**
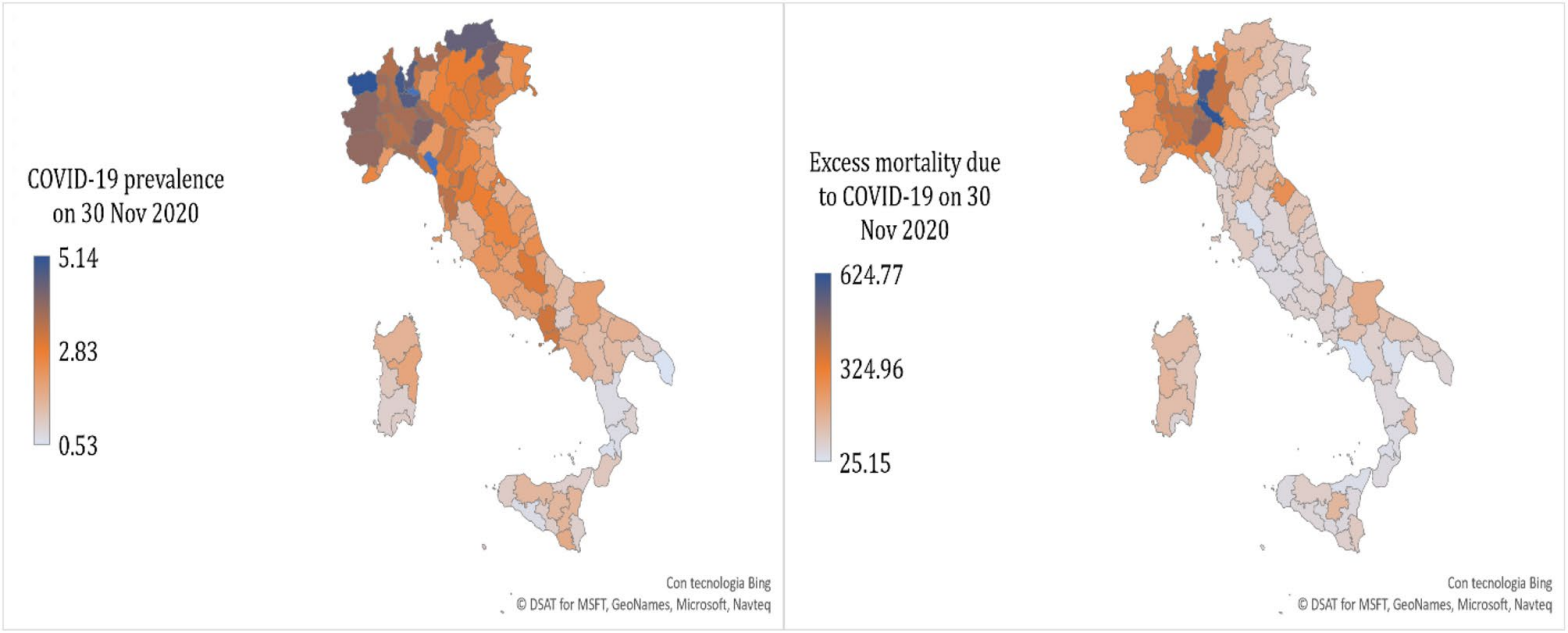
COVID-19 prevalence and related mortality in 107 Italian provinces (on 30 November 2020). Source: own elaborations on data from Italian Ministry of Health^109^, I.Stat^36^, and Istat^108^. The map was generated using Microsoft Excel software 2021.

As dependent variables, I use (i) the number of cumulative confirmed COVID-19 cases on 30 November 2020, in each province; (ii) the proportion of the total resident population infected by COVID-19 on 30 November 2020, in each province; and (iii) the difference, absolute and standardized for population size, between the number of deaths from all causes from March 2020 to November 2020, and the number of deaths from all causes in the March-November five-year average (from 2015 to 2019). [Note 4: Data on COVID-19 prevalence and excess mortality rate (on 30 November 2020) are graphically represented in Fig. 2].

Since the national number of excess deaths from all causes was exceptionally high in the period 1 March 2020–30 November 2020 (91,416), and equal to 11,427 excess deaths per month, many of them may be reasonable attributed to COVID-19^108^. The detailed definitions and the sources of all the independent and dependent variables used in this paper are reported in Table B1 (Appendix B). A summary of the main descriptive statistics is also provided in Table C1 (Appendix C).

## Empirical strategy

The main goal of this paper is to estimate the relationship between long-term exposure to nine air pollutants and COVID-19 transmissibility and mortality across 107 Italian provinces, using different econometric techniques. To measure the spread of COVID-19, I use both the absolute confirmed cases of the disease and its prevalence, expressed as a percentage of the population, as of 30 November 2020. This date was chosen by looking at the peak of the use of daily nasal swabs for testing COVID-19 at the second peak of the epidemic, which can be dated to the end of November 2020. In fact, at that time, more than 200,000 swabs were used daily^110^, and it is possible to hypothesize that they presented a reliable snapshot of reality. Although the number of daily swabs was even higher during the third wave of the COVID-19 epidemic, I preferred not to use these data. In fact, the third peak of the epidemic occurred around 8 April 2021, when more than 14% of the Italian population had received at least one dose of a COVID-19 vaccine^111^. Moreover, in a cross-section analysis the differences across units are more important than the number of infections. This choice may mitigate the inevitable bias in detecting infected people, which was also probably raised in early 2021 due to the start of the nationwide COVID-19 vaccination campaign.

Regarding the empirical strategy, I used a negative binomial regression that fits well when the dependent variable is a count variable, such as the COVID-19 cumulative confirmed cases and related deaths. [Note 5: In fact, the standard deviation exceeds the mean both for COVID-19 confirmed cases and related deaths (Table C1, Appendix C). In particular, the coefficient of variation, which is the ratio of the standard deviation to the mean, is 1.43 for COVID-19 cases and 1.54 for excess deaths, by suggesting a certain degree of variability in the dependent variables]. The choice of a negative binomial approach instead of a standard Poisson regression is based on the evaluation of the likelihood-ratio (LR) test on the overdispersion parameter alpha and is consistent with earlier similar studies on the same matter^14,18^. The negative binomial regression can be considered a generalization of Poisson regression that allows the conditional variance to exceed the conditional mean. To do this, the negative binomial approach considers an extra parameter that corrects the effects of the larger variance on the p-values^112^. To avoid biased results, I also include the size of the provincial population as an exposure variable. This is a pivotal point, because it allows to standardize the cumulative confirmed cases and excess deaths, that is, convert each observation from a count variable into a rate. As result, I estimate the following basic equation:

1 $${Covid}_{i}={\beta }_{0}+{\beta }_{1}{D}_{i}+{\beta }_{2}{DE}_{i}+{\beta }_{3}{E}_{i}+{\beta }_{4}{M}_{i}+{\beta }_{5}{Pollutant}_{i}+{\varepsilon }_{i},$$

where $i$ identifies each province, ${\beta }_{0}$ is a constant, ${D}_{i}$ is a vector of dummy variables for identifying Italian provinces with international borders, ${DE}_{i}$ is a vector of demographic and economic factors, ${E}_{i}$ is a vector of epidemiological features, ${M}_{i}$ is a vector of meteorological conditions, ${Pollutant}_{i}$ refers to the concentrations or violations of nine selected air pollutants (NO_2_, O_3_, PM_2.5_, PM_10_, benzene, BaP, As, Cd, and Ni), and ${\varepsilon }_{i}$ is the error term.

As sensitivity checks, I modeled the cases and deaths of COVID-19, using a standard ordinary least squares (OLS) approach and a spatial-autoregressive (SAR) framework. OLS can be considered the most widely used econometric technique for linear statistical models. It takes the same form of Eq. (1), with the only exception of the dependent variables, which are the prevalence and the excess mortality at the provincial level.

However, this procedure is not immune from issues, because from a theoretical point of view it is unlikely that neighboring provinces did not affect each other. In fact, the transmission within neighbor territories may have been affected by the movement of people, which is easier and faster across provinces’ borders. The presence of spatial dependence in the dependent variable may lead to substantial bias in OLS models^113^, resulting in inconsistent outcomes. Thus, I controlled for possible spatial effects in the dependent variable by following two sequential steps: (i) I investigated the map of COVID-19 prevalence on 30 November 2020 to make sure that an eventual spatial pattern was visible; and (ii) I calculated a common measure of spatial autocorrelation, the global Moran’s I statistic^114,115^, to verify whether each infection had the same likelihood of occurring at any location. Based on the evaluation of these metrics, I implemented a spatial-autoregressive model (SAR). In particular, the model was estimated with a maximum likelihood (ML) approach instead of the more common generalized spatial two-stage least squares (GS2SLS) approach. This choice is justified by performing Cameron and Trivedi’s^116^ decomposition of White’s information matrix (IM) test over the hypothesis of normality and heteroscedasticity of the errors, which needs to be met to implement the ML estimator [Ref.^117^, p. 236].

The equation estimated for the SAR model was eventually obtained by adding a spatially lagged dependent variable to the basic Eq. (1), that accounts for the endogenous interaction effects (2). The spatially lagged dependent variable aimed to verify if and how much a given province was influenced by the COVID-19 prevalence and excess mortality rate of the neighbor provinces. The final equation takes the following form:

2 $${Covid}_{i}={\beta }_{0}+{\beta }_{1}{D}_{i}+{\beta }_{2}{DE}_{i}+{\beta }_{3}{E}_{i}+{\beta }_{4}{{M}_{i}+\beta }_{5}{Pollutant}_{i}+{\rho w}_{i}{Covid}_{i}+{\varepsilon }_{i},$$

where $i$ identifies each province, ${\beta }_{0}$ is a constant, ${D}_{i}$ is a vector of dummy variables for identifying Italian provinces with international borders, ${DE}_{i}$ is a vector of demographic and economic characteristics, ${E}_{i}$ is a vector of epidemiological features, ${M}_{i}$ is a vector of meteorological conditions, ${Pollutant}_{i}$ refers to the average concentrations (or violations) of nine selected air pollutants (NO_2_, O_3_, PM_2.5_, PM_10_, Benzene, BaP, As, Cd, and Ni), ${\rho }_{i}$ is the spatially lagged dependent variable, ${w}_{i}$ is an inverse-distance weighted matrix with a 50 km cut-off, 75 km cut-off, 100 km cut-off, and no cut-off, and finally ${\varepsilon }_{i}$ is the error term. The matrix was row standardized because: (i) this allows for comparing spatial parameters that come from different models; and (ii) since all the weights summed to 1, the fact that one feature may have two neighbors, and another may have many more does not have a large effect on the results.

Finally, as a further sensitivity check, I used the data on COVID-19 prevalence rates and excess mortality on 28 February 2021, that is approximately one year after the start of the COVID-19 outbreak in Italy. This aimed to test whether the relationship between major air pollutants and COVID-19 spread, and related mortality was maintained over time.

## Results and discussion

### Negative binomial regressions

In Tables 6 and 7, I present the negative binomial model estimations for the Italian provinces. All models were significant; in fact, the Fisher-Snedecor distribution assumed values far higher than the tabulated critical values at the 1% level of significance. The McFadden’s^118^ pseudo-R^2^ is substantially homogenous across specifications and ranges between 0.07 and 0.09 for confirmed cases and from 0.07 and 0.1 for excess deaths. [Note 6: Although these values are low, it should be noted that pseudo-R^2^ values are usually much lower than those of the classic R-square^133^. However, OLS and SAR models are used to strengthen the results in “OLS regression models” and “Robustness checks: spatial-autoregressive analysis”, respectively.] Moreover, the likelihood-ratio (LR) chi-square test allows us to strongly reject the null hypothesis that the dispersion parameter alpha is equal to zero. Thus, the negative binomial approach is a better fit for the data than the Poisson regression.

**Table 6:** Results from negative binomial regressions on COVID-19 cumulative cases registered on 30 November 2020.

Variables	Model 1	Model 2	Model 3	Model 4	Model 5	Model 6
**Part A**						
AUT border	0.1157 [0.1928]	0.4794** [0.1946]	0.4919*** [0.1888]	0.5546*** [0.205]	0.4678** [0.1965]	0.5651*** [0.1865]
FRA border	0.2255* [0.1368]	0.5527*** [0.1174]	0.5433*** [0.1032]	0.5997*** [0.1147]	0.4872*** [0.1179]	0.4522*** [0.1153]
SLO border	− 0.0624 [0.2013]	0.0058 [0.2215]	− 0.1269 [0.2111]	− 0.154 [0.247]	− 0.0249 [0.2259]	0.0342 [0.2158]
SWI border	0.271** [0.1148]	0.385*** [0.1115]	0.4286*** [0.1015]	0.4212*** [0.1161]	0.3599*** [0.1127]	0.4465*** [0.1055]
Aged 0–19	− 0.0249 [0.0275]	− 0.0134 [0.0288]	− 0.0191 [0.0268]	− 0.0253 [0.0294]	0.0198 [0.0306]	0.0059 [0.0283]
Airport distance	− 0.0021** [0.0009]	− 0.0016* [0.0009]	− 0.0008 [0.0009]	− 0.0015 [0.001]	− 0.0009 [0.0009]	− 0.0015* [0.0009]
Foreigners	0.0443*** [0.0108]	0.0467*** [0.0113]	0.0414*** [0.0132]	0.0568*** [0.0145]	0.0439*** [0.0117]	0.0486*** [0.0109]
Male	0.0653 [0.0817]	0.1037 [0.0844]	0.0386 [0.0833]	0.1127 [0.0924]	− 0.0344 [0.0927]	0.0034 [0.085]
Pop. Density	0.0003*** [0.0001]	0.0001** [0.0001]	0.0002*** [0.0001]	0.0002*** [0.0001]	0.0002*** [0.0001]	0.0002*** [0.0001]
Urbanization	− 0.0396 [0.0523]	− 0.0841 [0.0579]	− 0.0686 [0.0535]	− 0.0127 [0.0688]	− 0.0488 [0.0571]	− 0.0835 [0.0549]
LRT disease	0.0014 [0.0041]	0.0056 [0.0439]	− 0.0029 [0.0039]	− 0.0003 [0.0044]	− 0.0002 [0.0045]	0.0028 [0.0042]
Large firms	− 0.0086 [0.0076]	− 0.0018 [0.0079]	− 0.0033 [0.0081]	− 0.0041 [0.0095]	− 0.0088 [0.0083]	− 0.0051 [0.0075]
Altitude	0.0001 [0.0002]	0.0004** [0.0002]	0.0003* [0.0002]	0.0003* [0.0002]	0.0006*** [0.0002]	0.0007*** [0.0002]
Rainy days	− 0.002 [0.003]	0.0025 [0.0029]	0.0004 [0.0027]	0.0032 [0.003]	0.0032 [0.0029]	0.0029 [0.0027]
Temperature	− 0.0873*** [0.0179]					
NO_2_		0.0132*** [0.0042]				
O_3 (>120)_			0.0071*** [0.0014]			
O_3 (>180)_				− 0.0002 [0.0017]		
PM_2.5_					0.0298*** [0.0069]	
PM_10_						0.0275*** [0.0058]
Pseudo R^2^	0.0737	0.0682	0.0823	0.0701	0.0711	0.0732
N	107	107	98	95	97	107
LR test (p.value)	0.0000	0.0000	0.0000	0.0000	0.0000	0.0000

Variables	Model 7	Model 8	Model 9	Model 10	Model 11	Model 12
**Part B**						
AUT border	0.5187*** [0.1928]	0.4071 [0.2737]	0.3981** [0.1892]	0.1653 [0.2591]	0.1134 [0.2355]	0.1039 [0.2449]
FRA border	0.449*** [0.1208]	0.7805*** [0.1358]	0.5101*** [0.1421]	0.6889*** [0.1384]	0.6866*** [0.1302]	0.6991*** [0.1396]
SLO border	0.0095 [0.2205]	− 0.1138 [0.2961]	− 0.1284 [0.2264]	0.008 [0.2671]	0.0392 [0.258]	0.063 [0.2539]
SWI border	0.4273*** [0.1086]	0.3844** [0.1584]	0.3427*** [0.1292]	0.3811*** [0.1265]	0.445*** [0.1189]	0.3963*** [0.1253]
Aged 0–19	0.0193 [0.0296]	0.0171 [0.0338]	− 0.087 [0.036]	− 0.0088 [0.0335]	0.0015 [0.0317]	− 0.001 [0.0338]
Airport distance	− 0.0015 [0.0009]	− 0.0008 [0.0011]	− 0.0007 [0.001]	0.0009 [0.0011]	0.0004 [0.001]	0.0013 [0.0011]
Foreigners	0.0479*** [0.0111]	0.0499*** [0.0129]	0.0497*** [0.0128]	0.0272 [0.0168]	0.0226 [0.0157]	0.0269 [0.0166]
Male	− 0.0158 [0.0879]	0.022 [0.0994]	0.0837 [0.1044]	− 0.122 [0.1061]	− 0.1235 [0.0986]	− 0.0924 [0.1032]
Pop. Density	0.0002*** [0.0001]	0.0002** [0.0001]	0.0001 [0.0001]	0.0000 [0.0001]	0.0000 [0.0001]	0.0000 [0.0001]
Urbanization	− 0.0839 [0.0563]	− 0.0997 [0.0653]	0.0556 [0.0622]	− 0.0102 [0.064]	− 0.0179 [0.0604]	0.0023 [0.0648]
LRT disease	0.0011 [0.0042]	− 0.0026 [0.0046]	− 0.0044 [0.0049]	− 0.0005 [0.0056]	0.0026 [0.0053]	− 0.0004 [0.0055]
Large firms	− 0.0089 [0.0079]	0.0009 [0.0089]	0.0198** [0.01]	0.0275** [0.0129]	0.0358*** [0.0124]	0.0269** [0.0129]
Altitude	0.0005*** [0.0002]	0.0002 [0.0002]	0.0006*** [0.0002]	0.0001 [0.0002]	0.0001 [0.0002]	0.0001 [0.0002]
Rainy days	0.0029 [0.0027]	0.0053 [0.0032]	− 0.0019 [0.0034]	0.0000 [0.0037]	0.0005 [0.0035]	− 0.0009 [0.0037]
PM_10 (>50)_	0.0058*** [0.0014]					
Benzene		0.1317** [0.0641]				
BaP			0.0832 [0.0881]			
As				0.0504 [0.0525]		
Cd					0.197*** [0.0694]	
Ni						− 0.0154 [0.0174]
Pseudo R^2^	0.0709	0.0682	0.0847	0.0847	0.0898	0.0846
N	107	88	73	60	60	60
LR test (p.value)	0.0000	0.0000	0.0000	0.0000	0.0000	0.0000

p-value < 0.01***; p-value < 0.05**; p-value < 0.1*. Standard errors in parentheses. All models included a constant, a dummy for regional capitals, and controls for the size of the province, smokers, and obese individuals.

**Table 7:** Results from negative binomial regressions on COVID-19 cumulative excess deaths registered on 30 November 2020.

Variables	Model 1	Model 2	Model 3	Model 4	Model 5	Model 6
**Part A**						
AUT border	− 0.6471 [0.3953]	0.2168 [0.3946]	0.3104 [0.3556]	0.2357 [0.3916]	0.124 [0.385]	0.29 [0.4045]
FRA border	− 0.4053 [0.2539]	0.2524 [0.2215]	0.3561** [0.1802]	0.5923*** [0.182]	0.1483 [0.2061]	0.2821 [0.2207]
SLO border	− 0.4116 [0.4021]	− 0.192 [0.4207]	− 0.386 [0.3888]	− 0.1852 [0.4642]	− 0.1785 [0.4114]	− 0.1389 [0.4293]
SWI border	− 0.3048 [0.2139]	− 0.1652 [0.2109]	− 0.0103 [0.1714]	0.1914 [0.1821]	− 0.2937 [0.2005]	− 0.01 [0.2088]
Aged 0–19	− 0.0614 [0.0516]	− 0.0528 [0.0534]	− 0.0373 [0.049]	− 0.0606 [0.05]	0.0244 [0.0535]	− 0.0276 [0.0552]
Airport distance	− 0.0019 [0.0017]	− 0.0005 [0.0017]	0.0012 [0.0015]	0.001 [0.0016]	0.0007 [0.0017]	− 0.0003 [0.0018]
Foreigners	0.0185 [0.0201]	0.0216 [0.0219]	0.0138 [0.0236]	0.0387 [0.0251]	0.0082 [0.0223]	0.0234 [0.0225]
Male	0.7309*** [0.1432]	0.791*** [0.1468]	0.5838*** [0.1429]	0.5059*** [0.1494]	0.553*** [0.1512]	0.6632*** [0.1525]
Pop. Density	0.0001 [0.0001]	− 0.0003** [0.0001]	− 0.0001 [0.0001]	− 0.0000 [0.0001]	− 0.0003** [0.0001]	− 0.0002 [0.0001]
Urbanization	0.2173** [0.0905]	0.1714* [0.0985]	0.1775** [0.0875]	− 0.0165 [0.1073]	0.2939*** [0.0952]	0.2056** [0.0999]
LRT disease	0.0285*** [0.0073]	0.0252*** [0.0079]	0.0179*** [0.0066]	0.0234*** [0.0067]	0.033*** [0.0081]	0.0241*** [0.008]
Large firms	0.0073 [0.0145]	0.0282* [0.0161]	0.0156 [0.0145]	0.0279 [0.0172]	0.0217 [0.0162]	0.0289* [0.0162]
Altitude	− 0.0001 [0.0003]	0.0005* [0.0003]	0.0001 [0.0003]	− 0.0002 [0.0003]	0.0006** [0.0003]	0.0006* [0.0003]
Rainy days	− 0.0208*** [0.0057]	− 0.102* [0.0054]	− 0.0107** [0.0048]	− 0.0112** [0.0054]	− 0.0076 [0.0055]	− 0.0088 [0.0055]
Temperature	− 0.1857*** [0.0339]					
NO_2_		0.0244*** [0.0077]				
O_3 (>120)_			0.0175*** [0.0024]			
O_3 (>180)_				0.0152*** [0.0024]		
PM_2.5_					0.0319** [0.0125]	
PM_10_						0.0274** [0.0108]
Pseudo R^2^	0.0793	0.0697	0.0964	0.0922	0.0744	0.0675
N	107	107	98	95	97	107
LR test (p.value)	0.0000	0.0000	0.0000	0.0000	0.0000	0.0000

Variables	Model 7	Model 8	Model 9	Model 10	Model 11	Model 12
**Part B**						
AUT border	0.2207 [0.4127]	0.1848 [0.5306]	0.062 [0.4212]	0.1716 [0.4615]	− 0.2905 [0.4983]	− 0.2925 [0.5004]
FRA border	0.3053 [0.2263]	0.3774 [0.2403]	0.4083 [0.2579]	0.1052 [0.2254]	0.0089 [0.2341]	0.0139 [0.2375]
SLO border	− 0.1984 [0.4318]	− 0.0988 [0.5383]	− 0.073 [0.4645]	− 0.0211 [0.4808]	0.1759 [0.4796]	0.1627 [0.4724]
SWI border	− 0.0275 [0.2122]	− 0.2048 [0.2742]	− 0.048 [0.2338]	− 0.5093** [0.1987]	− 0.4634** [0.2239]	− 0.4672** [0.2218]
Aged 0–19	− 0.0153 [0.0562]	− 0.0359 [0.0621]	− 0.14** [0.0667]	− 0.0818 [0.0571]	− 0.0347 [0.0598]	− 0.0344 [0.0602]
Airport distance	− 0.0002 [0.0018]	− 0.001 [0.0021]	0.0011 [0.0017]	0.0004 [0.0018]	0.0005 [0.0021]	0.0007 [0.0022]
Foreigners	0.0299 [0.0226]	0.0393 [0.0241]	0.0125 [0.0247]	0.0337 [0.0306]	0.0274 [0.0329]	0.0291 [0.0334]
Male	0.6392*** [0.1551]	0.7007*** [0.1701]	0.7442*** [0.1771]	0.4656*** [0.1594]	0.6479*** [0.1661]	0.6556*** [0.1695]
Pop. Density	− 0.0002 [0.0001]	− 0.0003* [0.0001]	− 0.0007*** [0.0002]	− 0.0005*** [0.0002]	− 0.0004*** [0.0002]	− 0.0004*** [0.0002]
Urbanization	0.2083** [0.1007]	0.2136** [0.1082]	0.4001*** [0.1054]	0.3728*** [0.1062]	0.3635*** [0.1148]	0.3705*** [0.1202]
LRT disease	0.0224*** [0.008]	0.0201** [0.0082]	0.0038 [0.0098]	0.0216** [0.0093]	0.024** [0.0101]	0.0236** [0.0099]
Large firms	0.0266 [0.0167]	0.0314* [0.0174]	0.0742*** [0.0186]	0.0472** [0.0204]	0.0449** [0.0227]	0.0432* [0.0221]
Altitude	0.0004 [0.0003]	0.0007* [0.0004]	0.0006* [0.0003]	0.0012*** [0.0004]	0.0013*** [0.0004]	0.0013*** [0.0004]
Rainy days	− 0.0084 [0.0055]	− 0.0107* [0.0061]	− 0.0162** [0.0064]	− 0.0175*** [0.0065]	− 0.0223*** [0.007]	− 0.0226*** [0.0073]
PM_10 (>50)_	0.0051* [0.0026]					
Benzene		0.1152 [0.1218]				
BaP			− 0.5869*** [0.1785]			
As				0.3309*** [0.0841]		
Cd					0.0256 [0.1484]	
Ni						− 0.0045 [0.0325]
Pseudo R^2^	0.066	0.0722	0.0876	0.1006	0.0876	0.0875
N	107	88	73	60	60	60
LR test (p. value)	0.000	0.0000	0.0000	0.0000	0.000	0.000

p-value < 0.01***; p-value < 0.05**; p-value < 0.1*. Standard errors in parentheses. All models included a constant, a dummy for regional capitals, and controls for the size of the province, smokers, and obese individuals.

Regarding control variables, the results showed that a border with Austria, France, and Switzerland, the share of foreigners, population density, and altitude were significantly and positively correlated with cumulative confirmed COVID-19 cases on 30 November 2020 (Table 6). [Note 7: The meaning of the relationship between control variables and COVID-19 cases and deaths will be explained in the next “OLS regression models”]. Conversely, distance from the nearest main airport and average temperature were significantly and negatively associated with total confirmed COVID-19 cases. Regarding air pollutants, NO_2_, O_3(>120)_, PM_2.5_, PM_10_, benzene, and Cd showed a positive and statistically significant relationship with COVID-19 infections. For the remainder, BaP, As, and Ni were not significant at all.

Since coefficients that come from negative binomial models cannot easily be interpreted, I computed the marginal effect for the air pollutants that were statistically significant (Table 8). The most significant coefficients for COVID-19 cases were NO_2_, O_3(>120)_, PM_2.5_ PM_10_, and Cd, which were verified at 1% level of significance, followed by benzene which was verified at 5% level of significance. Regarding primary pollutants, 1 μg/m^3^ increase in PM_2.5_, PM_10_, and NO_2_ concentrations was associated with average increases of 463.2, 405, and 194.2 COVID-19 infections respectively, while for PAHs and heavy metals, a 0.1 μg/m^3^ increase in benzene and a 0.1 ng/m^3^ increase in Cd was associated with average increment of 211.6 and 366.7 COVID-19 infections, respectively. [Note 8: I chose 0.1 units for benzene and BaP because their legal threshold was comparatively much lower than that for common air pollutants]. Thus, among common air pollutants, PM_2.5_ and PM_10_ seemed to have the most adverse effects on COVID-19 spread, while Cd was the most dangerous among the remaining pollutants.

**Table 8:** The average marginal effects got from negative binomial regressions.

Cases	NO_2_	O_3_	PM_2.5_	PM_10_	Benzene	Cd
	1 µg/m^3^	> 120 µg/m^3^	1 µg/m^3^	1 µg/m^3^	0.1 µg/m^3^	0.1 ng/m^3^
Marginal effects	194.18*** [61.84]	108.26*** [21.49]	463.23*** [107.2]	405.01*** [85.54]	211.6** [103.2]	366.72*** [129.44]
95% CI	72.98–315.38	66.14–150.36	253.13–673.34	237.35–572.66	9.33–413.86	113.03–620.42

Deaths	NO_2_ 1 µg/m^3^	O_3_ > 120 µg/m^3^	PM_2.5_ 1 µg/m^3^	PM_10_ 1 µg/m^3^	BaP 0.1 ng/m^3^	As 0.1 ng/m^3^
Marginal effects	20.8*** [6.62]	15.69*** [2.22]	29.27** [11.52]	23.44** [9.25]	− 56.99*** [17.49]	37.7*** [9.7]
95% CI	7.82–33.79	11.34–20.03	6.7–51.85	5.32–41.56	− 91.27 to − 22.71	18.69–56.72

*CI* confidence interval. p-value < 0.01***; p-value < 0.05**. Standards errors in parentheses.

With regards to COVID-19 related deaths, male population, urbanization, large firms, LRT disease, and altitude (although barely) were positively and significantly correlated with the excess deaths. Conversely, population density, rainy days, and temperature were negatively and significantly correlated with the excess deaths (Table 7).

The most significant air pollutants were NO_2_, O_3(>120)_, O_3(>180)_, Bap, and As, which were verified at 1% level of significance, followed by PM_2.5_ and PM_10_, which were verified at 5% level of significance. Marginal effects (in Table 8) showed that a 1 μg/m^3^ increase in PM_2.5_, PM_10_, and NO_2_ concentrations was correlated with an average increase of 29.3, 23.4, and 20.8 COVID-19 related deaths, respectively. For the remaining, a 0.1 ng/m^3^ in As was associated with an average increment of 37.7 COVID-19 related deaths, while a 0.1 ng/m^3^ increase in BaP was correlated with an average decrease of 57 COVID-19 related deaths. Thus, As, PM_2.5_, and PM_10_ showed the largest positive effect on COVID-19 related deaths.

### OLS regression models

To strengthen the results, in Tables 9 and 10, I estimated an OLS regression model for COVID-19 prevalence and excess mortality in the Italian provinces. Since standard errors are usually biased in small samples, I corrected them for heteroscedasticity by applying the HC2 estimator proposed by MacKinnon and White^119^, which performs well even when sample size is not large [Ref.^120^, p. 533]. The Fisher–Snedecor distribution was highly significant and verified at a 1% level of significance for all the OLS models; therefore, the choice of the independent variables can be justified. In Tables D1 and D2 (Appendix D), I also report the Cameron and Trivedi’s^116^ decomposition of IM-test for heteroscedasticity, skewness, and kurtosis. The tests show that the null hypothesis can be safely accepted in all models, i.e., the residuals were homoscedastic and normally distributed. [Note 9: It is necessary to stress that in model 5 (excess mortality), the null hypothesis of residuals normality was rejected (Table D2, Appendix D). However, it does not seem matter of concern because the histogram of the residuals suggests that distribution of residuals was not skewed (Fig. D3, Appendix D)]. Moreover, the R-square ranged from 0.73 to 0.81 for prevalence, and from 0.39 to 0.62 for excess mortality. Thus, the models were a good fit and explained a large and moderate fraction of the variability of COVID-19 prevalence and related mortality, respectively. The variance inflation factors (VIF) were always less than the threshold of 5, suggesting that there were no severe multicollinearity issues^121^. The only exception was the coefficient of the temperature in model 1, which was carefully excluded by the other models.

**Table 9:** Results from OLS models on COVID-19 prevalence rate registered on 30 November 2020.

Variables	Model 1	Model 2	Model 3	Model 4	Model 5	Model 6
**Part A**						
AUT border	0.5526 [0.5076]	1.0847** [0.4834]	1.2464** [0.5222]	1.2347*** [0.4552]	0.9257* [0.4847]	1.2091*** [0.4476]
FRA border	0.2765 [0.3859]	0.8118** [0.3512]	0.8716** [0.3854]	0.8567* [0.4638]	0.6882* [0.3879]	0.7548** [0.3382]
SLO border	− 0.3496 [0.2308]	− 0.1202 [0.2803]	− 0.3594 [0.3505]	− 0.5464 [0.3983]	− 0.0606 [0.3331]	− 0.0073 [0.3379]
SWI border	0.6864* [0.4074]	1.1821*** [0.2398]	1.2889*** [0.2571]	1.3146*** [0.2488]	1.0829*** [0.2346]	1.3433*** [0.2171]
Aged 0–19	− 0.046 [0.0545]	− 0.0457 [0.0611]	− 0.0558 [0.0486]	− 0.0669 [0.0543]	0.0133 [0.0657]	− 0.0434 [0.0557]
Airport distance	− 0.0041** [0.0018]	− 0.0033* [0.0019]	− 0.0032* [0.0017]	− 0.0038** [0.0017]	− 0.0021 [0.0018]	− 0.003 [0.0018]
Foreigners	0.1027*** [0.0238]	0.1033*** [0.0223]	0.1008*** [0.03]	0.1194*** [0.0303]	0.0936*** [0.024]	0.1051*** [0.0227]
Male	0.2845* [0.1706]	0.3136 [0.1941]	0.2192 [0.1616]	0.29 [0.1833]	0.1054 [0.2097]	0.168 [0.1699]
Pop. density	0.001*** [0.0002]	0.0006*** [0.0002]	0.0007*** [0.0002]	0.0007*** [0.0002]	0.0006*** [0.0002]	0.0007*** [0.0002]
Urbanization	0.099 [0.1119]	0.0043 [0.1209]	0.038 [0.1268]	0.0286 [0.1353]	0.0642 [0.1256]	0.0448 [0.1079]
LRT disease	0.0177* [0.0093]	0.0173* [0.0094]	0.0116 [0.0096]	0.0144 [0.0097]	0.018 [0.0126]	0.0169* [0.0101]
Large firms	0.0079 [0.0216]	0.0425** [0.0211]	0.0345 [0.0224]	0.0587* [0.0301]	0.0284 [0.0224]	0.0267 [0.0191]
Altitude	0.0006** [0.0003]	0.0012*** [0.0003]	0.001*** [0.0003]	0.001*** [0.0003]	0.0013*** [0.0003]	0.0014*** [0.0003]
Rainy days	− 0.009 [0.0073]	− 0.0025 [0.0069]	− 0.0032 [0.0053]	− 0.0003 [0.0057]	0.0001 [0.0064]	0.0008 [0.006]
Temperature	− 0.1781*** [0.0533]					
NO_2_		0.0265*** [0.0095]				
O_3 (>120)_			0.0114*** [0.0042]			
O_3 (>180)_				0.0008 [0.0068]		
PM_2.5_					0.0438*** [0.0141]	
PM_10_						0.0537*** [0.0111]
Adjusted R^2^	0.7436	0.7268	0.7832	0.7554	0.727	0.7491
N	107	107	98	95	97	107
F-test	21.02***	20.03***	24.63***	20.75***	24.93***	28.96***
VIF (range)	1.38–5.55	1.32–2.51	1.34–3.02	1.36–2.92	1.38–2.58	1.33–2.6

OLS 30 Nov prevalence	Model 7	Model 8	Model 9	Model 10	Model 11	Model 12
**Part B**						
AUT border	1.2309** [0.4721]	0.4444 [0.3607]	1.2065*** [0.3113]	0.2496 [0.4786]	0.2034 [0.403]	0.1013 [0.4998]
FRA border	0.7619** [0.3329]	1.3877*** [0.269]	1.1103*** [0.2097]	1.4495*** [0.2571]	1.4075*** [0.2719]	1.4799*** [0.3018]
SLO border	− 0.1375 [0.3255]	− 0.1433 [0.3059]	− 0.5125* [0.2911]	− 0.0032 [0.3415]	0.0569 [0.3355]	0.0737 [0.3872]
SWI border	1.2652*** [0.227]	1.2806*** [0.2154]	0.9622*** [0.3138]	1.2868*** [0.2741]	1.4586*** [0.2396]	1.3364*** [0.2599]
Aged 0–19	− 0.0228 [0.0578]	0.0273 [0.0578]	− 0.1473** [0.0588]	0.0206 [0.064]	0.0176 [0.0608]	0.045 [0.0665]
Airport distance	− 0.0027 [0.0019]	− 0.0022 [0.0021]	− 0.0019 [0.0017]	0.0018 [0.0021]	0.0005 [0.0021]	0.0026 [0.0024]
Foreigners	0.1038*** [0.0233]	0.1071*** [0.0258]	0.1074*** [0.0224]	0.0699** [0.0317]	0.0637** [0.0311]	0.0732** [0.0349]
Male	0.137 [0.1769]	0.0886 [0.1838]	0.3569 [0.2285]	− 0.2063 [0.2269]	− 0.1369 [0.2097]	− 0.2248 [0.2252]
Pop. density	0.0007*** [0.0002]	0.0006*** [0.0002]	0.0008* [0.0004]	0.0006 [0.0004]	0.0007* [0.0004]	0.0007* [0.0004]
Urbanization	0.0586 [0.1131]	− 0.1302 [0.1246]	0.1479 [0.1455]	− 0.0106 [0.1632]	− 0.0775 [0.1569]	− 0.0182 [0.1538]
LRT disease	0.0144 [0.0103]	0.0108 [0.0095]	0.0139 [0.0123]	0.0124 [0.0124]	0.0172 [0.0118]	0.0104 [0.012]
Large firms	0.0231 [0.0205]	0.056** [0.0241]	0.066** [0.0258]	0.079** [0.0347]	0.0939*** [0.0348]	0.0712* [0.0359]
Altitude	0.0013*** [0.0003]	0.0008** [0.0004]	0.0015*** [0.0003]	0.0005 [0.0004]	0.0006 [0.0004]	0.0005 [0.0004]
Rainy days	− 0.0001 [0.0059]	0.0029 [0.0069]	− 0.0079 [0.0059]	− 0.0031 [0.0065]	− 0.0026 [0.0064]	− 0.0044 [0.0066]
PM_10 (>50)_	0.0121*** [0.0028]					
Benzene		0.3023*** [0.1118]				
BaP			0.2352 [0.1893]			
As				0.0984 [0.1565]		
Cd					0.4211* [0.232]	
Ni						− 0.0205 [0.0252]
Adjusted R^2^	0.7409	0.7404	0.8055	0.7926	0.8099	0.7913
N	107	88	73	60	60	60
F-test	24.5***	33.63***	49.79***	46.12***	35.91***	27.47***
VIF (range)	1.33–2.6	1.44–2.69	1.53–3.45	1.65–3.94	1.56–4.17	1.65–4.2

p-value < 0.01***; p-value < 0.05**; p-value < 0.1*. Standard errors in parentheses. All models included a constant, a dummy for regional capitals, and controls for the size of the province, smokers, and obese individuals.

**Table 10:** Results from OLS models on COVID-19 excess mortality registered on 30 November 2020.

OLS excess rate	Model 1	Model 2	Model 3	Model 4	Model 5	Model 6
**Part A**						
AUT border	− 111.3462 [69.768]	− 38.4385 [61.3748]	− 9.3204 [43.8549]	− 15.6083 [42.4402]	− 55.1127 [53.2371]	− 19.5649 [46.0168]
FRA border	− 45.6631 [41.0405]	26.8474 [35.9419]	47.4902 [33.4588]	64.6251 [41.1535]	− 2.286 [43.7756]	18.1862 [35.2973]
SLO border	− 79.3963 [65.4272]	− 45.0156 [60.1806]	− 80.4848 [51.529]	− 31.6575 [32.3944]	− 49.1281 [54.2077]	− 27.8524 [43.8128]
SWI border	− 56.7891 [45.1511]	9.4731 [44.7109]	13.866 [43.8135]	19.9458 [53.9563]	− 15.4593 [45.5373]	33.9219 [44.2952]
Aged 0–19	− 7.58 [6.7072]	− 7.6384 [7.5525]	− 5.073 [6.1898]	− 5.2 [6.3734]	0.3553 [8.5625]	− 7.2876 [6.9699]
Airport distance	− 0.0527 [0.277]	0.0449 [0.2803]	0.1069 [0.2436]	0.0838 [0.2467]	0.1661 [0.2876]	0.104 [0.259]
Foreigners	1.3476 [4.7519]	1.2801 [4.5556]	− 1.3764 [4.799]	2.7003 [5.1123]	− 0.1322 [5.0285]	1.5438 [4.482]
Male	91.5918*** [29.4946]	96.1261*** [31.5021]	65.9252** [25.159]	47.8864* [24.557]	74.2964** [35.3288]	74.0357*** [27.2692]
Pop. Density	0.0196 [0.0253]	− 0.0317 [0.0341]	− 0.0175 [0.0216]	− 0.0258 [0.0435]	− 0.033 [0.0358]	− 0.0203 [0.0314]
Urbanization	31.3395 [20.9488]	17.3113 [22.1948]	22.6372 [19.86]	− 5.2991 [19.6849]	36.7292 [22.3791]	23.4627 [19.7158]
LRT disease	3.832** [1.7428]	3.8436** [1.7539]	2.6396 [1.7071]	3.3762** [1.5664]	5.1043** [2.1938]	3.7884** [1.8443]
Large firms	5.3308 [3.9811]	9.8938** [3.93]	4.1161 [4.4703]	6.8305 [5.4828]	7.3365* [4.1307]	7.4993* [3.8212]
Altitude	− 0.0252 [0.0585]	0.068 [0.0532]	0.0187 [0.0594]	0.0039 [0.0658]	0.0858 [0.0645]	0.1001* [0.0554]
Rainy days	− 2.6834*** [0.935]	− 1.8395** [0.851]	− 1.654** [0.8126]	− 1.5147* [0.7698]	− 1.3094 [0.8903]	− 1.3396 [0.8428]
Temperature	− 24.3919*** [6.5761]					
NO_2_		4.0154*** [1.2736]				
O_3 (>120)_			3.1558*** [0.5553]			
O_3 (>180)_				3.5981*** [0.9437]		
PM_2.5_					6.3702** [2.4974]	
PM_10_						8.156*** [2.2918]
Adjusted R^2^	0.3796	0.3625	0.5026	0.5373	0.3929	0.4062
N	107	107	98	95	97	107
F-test	9.31***	4.69***	8.23***	6.55***	5.15***	6.52***
VIF (range)	1.38–5.55	1.32–2.51	1.34–3.02	1.36–2.92	1.38–2.58	1.33–2.6

Variables	Model 7	Model 8	Model 9	Model 10	Model 11	Model 12
**Part B**						
AUT border	− 15.8608 [50.4814]	− 56.0389 [101.7693]	− 49.776 [50.8219]	− 26.1457 [92.9584]	− 107.7308 [123.5102]	− 109.6417 [124.8275]
FRA border	18.9963 [35.7498]	24.1802 [59.726]	45.4798 [65.2261]	26.2888 [56.8109]	6.6584 [62.4633]	7.8836 [71.0215]
SLO border	− 47.1256 [46.9817]	− 34.7954 [92.4303]	− 29.0193 [53.5155]	− 29.3902 [68.7289]	5.9243 [113.0937]	6.2294 [114.5465]
SWI border	22.0068 [43.639]	4.676 [52.935]	21.8521 [40.8342]	− 54.4474 [50.3919]	− 33.5368 [49.7223]	− 35.793 [49.4532]
Aged 0–19	− 4.1086 [7.0644]	− 4.4544 [8.1073]	− 16.3419* [9.0792]	− 11.5499 [9.2091]	− 4.4723 [10.7455]	− 3.9838 [11.0619]
Airport distance	0.1385 [0.2597]	− 0.1583 [0.4105]	0.0023 [0.3188]	0.1203 [0.4447]	0.2281 [0.4866]	0.2658 [0.5351]
Foreigners	1.3276 [4.7086]	5.9286 [5.8527]	− 0.3243 [3.7839]	3.21 [5.5548]	2.578 [5.8346]	2.7449 [6.17]
Male	68.9701** [27.2019]	85.6356** [34.9703]	89.0109** [43.7239]	52.7107 [41.7637]	48.8835 [44.0597]	47.2827 [47.217]
Pop. density	− 0.024 [0.0347]	− 0.0221 [0.0442]	− 0.0757 [0.0481]	− 0.0754 [0.052]	− 0.0533 [0.0489]	− 0.0532 [0.0517]
Urbanization	25.501 [19.7826]	14.6159 [26.3163]	40.5965 [31.5119]	45.4256 [33.1442]	37.8147 [36.0698]	38.8936 [34.0508]
LRT disease	3.4059* [1.8105]	3.6085* [2.0042]	1.4266 [1.8781]	2.964 [2.1372]	2.3063 [2.1887]	2.1827 [2.0975]
Large firms	6.8713* [4.0807]	9.2882* [4.8828]	15.1763*** [3.616]	12.7669** [5.1743]	11.9376** [5.2257]	11.5296** [5.5128]
Altitude	0.0738 [0.0553]	0.1017 [0.0751]	0.0993 [0.0631]	0.1472* [0.0801]	0.1516* [0.0842]	0.1506* [0.0835]
Rainy days	− 1.4729* [0.8333]	− 1.5213 [1.0526]	− 1.6786* [0.9471]	− 2.2064** [0.9851]	− 2.4453** [1.0466]	− 2.4768** [1.0894]
PM_10 (>50)_	1.8645*** [0.5762]					
Benzene		25.8048 [21.4386]				
BaP			− 59.3584* [32.9484]			
As				47.0851** [17.971]		
Cd					7.7207 [29.8248]	
Ni						− 0.346 [5.9456]
Adjusted R^2^	0.393	0.3421	0.376	0.4043	0.3284	0.3277
N	107	88	73	60	60	60
F-test	6.27***	4.28***	5.66***	7.53***	8.72***	7.86***
VIF (range)	1.33–2.6	1.44–2.69	1.53–3.45	1.65–3.94	1.56–4.17	1.65–4.2

p-value < 0.01***; p-value < 0.05**; p-value < 0.1*. Standard errors in parentheses. All models included a constant, a dummy for regional capitals, and controls for the size of the province, smokers, and obese individuals.

The results are similar to those obtained from the negative binomial regression models. Concerning the control variables, a border with Austria, France, and Switzerland, foreign population, population density, deaths from respiratory disease, and altitude were significantly and positively correlated with COVID-19 prevalence; meanwhile, distance from the nearest airport, and temperature were significantly and negatively associated with infection rates (Table 9). [Note 10: Obesity had an unexpected negative and significant association with COVID-19 prevalence, while smokers were not significant at all]. Notably, the coefficient of border with Switzerland was more significant and larger than those for border with Austria and Slovenia. This may be due to the flow of the 65,000 cross-border workers who reside in Italy and work in Switzerland, and who account for a total of 63.73% of all Italian cross-border commuters [Ref.^122^, pp. 184–185]. The significance of foreign population could be explained by foreigners’ greater propensity to travel to their native countries, which could have increased the probability of meeting infected people.

The direction of the correlation between population density and COVID-19 cases is consistent with recent literature^123,124^, suggesting the importance of keeping a safe physical distance from others to limit the spread of the outbreak. The positive significance of altitude, conversely, is in contrast with most of the recent literature^97,125–127^. However, these studies mainly focused on Latin American countries, such as Colombia, Peru, and Brazil, which have cities with altitude differences of up to more than 3000 m. As shown by Table C1 (Appendix C), the difference between the most low-altitude city (Venice) and the most high-altitude city (L’Aquila) is just 1167.3 m, suggesting a lower isolation of the population. Moreover, the size of the regression coefficient of altitude is extremely low. The positive effect of the prevalence of deaths from respiratory diseases in the period 2014–2019 seems to stress the greatest vulnerability of people with comorbidities, who are more likely to get infected^87,128^.

On the contrary, higher temperatures may have favored a reduction of COVID-19 transmission, and this result appears consistent with several recent studies^102,103,129–131^. The negative relationship between transmission and distance from the nearest airport seems to advocate the beneficial effect of travel restrictions.

Regarding air pollutants, NO_2_, O_3(>120)_, PM_2.5_, PM_10_, PM_10 (>50)_, and benzene were statistically significant at the 1% level, while Cd showed a significance level of 10% (Table 9). Among common air pollutants, 10 μg/m^3^ increases in the concentrations of NO_2_, PM_2.5_, and PM_10_ were associated respectively with average increments of 0.27% (95% CI 0.08–0.45), 0.44% (95% CI 0.16–0.72), and 0.54% (95% CI 0.32–0.76) of COVID-19 prevalence. Among significant PAHs and heavy metals, a 1-unit µg/m^3^ increase in benzene and a 1-unit ng/m^3^ increase in Cd was associated respectively with increments of 0.3% (95% CI 0.08–0.53) and 0.42% (95% CI − 0.05 to 0.89) in nationwide COVID-19 prevalence [Note 11: 95% CI stands per 95% confidence interval]. Thus, PM_10_ exhibited the largest dangerous effect on COVID-19 spread.

For the model 1–12 (Table 10), the results showed that, among control variables male population, LRT disease, and big firms were significantly and positively correlated with COVID-19 excess mortality. By the contrary, rainy days and temperature were significantly and negatively associated with COVID-19 excess mortality. [Note 12: Obesity and smokers had a negative and significant association with excess mortality rate. The virtuous impact of smoking seems to confirm the existence of a smokers’ paradox in COVID-19^96^. However, since data on obesity and smokers are available only at regional level, these outcomes should be treated with caution]. The adverse impact of COVID-19 disease on male population is large and consistent with other studies^77,78^. [Note 13: In fact, a 1-unit % increase in male population was associated with an increase up to 96 excess deaths per 100,000 people]. The positive effect of LRT deaths on COVID-19 excess mortality stresses the importance of comorbidities on COVID-19 patients outcomes^85–88^. The positive relationship between big firms and excess mortality due to COVID-19 seems to reinforce the idea that ambient air pollution can increase the severity of the disease. While the virtuous effect of historical rainy days can be explained considering the arguments put forward by Shenoy et al.^107^, which argued that rainfall may lead to higher social distancing. This could have mitigated the negative impact of the outbreak. The beneficial impact of higher temperatures is consistent with the literature^18,103–105^.

Regarding the air pollutants, NO_2_, O_3(>120)_, O_3(>180)_, PM_10_, and PM_10 (>50)_ were statistically significant at the 1% level of significance, while PM_2.5_ and As were verified at the 5% level of significance (Table 10). In particular, a 10 μg/m^3^ increase in the concentrations of NO_2_, PM_2.5_, and PM_10_ was associated with an average increment of 40.2 (95% CI 14.8–65.5), 63.7 (95% CI 14–113.4), and 81.6 (95% CI 36–127.1) excess deaths per 100,000 people, respectively. Among the remaining air pollutants, a 1-unit ng/m^3^ increase in As concentration was correlated with an average increment of 47.1 (95% CI 10.8–83.4) excess deaths per 100,000 people. Notwithstanding the BaP had an unexpected negative impact on COVID-19 excess mortality, it was only verified at 10% level of significance. Thus, the results confirm the adverse impact of outdoor air pollution on COVID-19 spread and mortality.

### Robustness checks: Spatial-autoregressive analysis

Tables 11 and 12 I presented the results of the SAR models on COVID-19 prevalence and excess mortality, on 30 November 2020, respectively. The use of the SAR approach is justified by the global Moran’s I, which allowed to reject the null hypothesis that data were randomly distributed both for the dependent and main independent variables. It ranges from − 1 (dispersion) to 1 (clustering). Specifically, the global Moran’s I was always positive and statistically significant at 1% level of confidence. Since the prevalence and excess mortality on November 2020 showed a Moran’s I of 0.341 and 0.362, they both were positively spatially correlated. That means that the high (HH) or low (LL) values of prevalence and excess mortality tended to be clustered spatially (Table E1, Appendix E).

**Table 11:** Results from SAR models on COVID-19 prevalence rate registered on 30 November 2020.

Prevalence	NO_2_	O_3_	PM_2.5_	PM_10_	Benzene	BaP	As	Cd	Ni
**50 km cut-off**									
Coefficient	0.024*** [0.008]	0.0112*** [0.0036]	0.035** [0.0141]	0.0487*** [0.012]	0.2546** [0.1264]	0.2188 [0.1693]	0.0681 [0.1087]	0.3928** [0.1719]	− 0.0289 [0.029]
Spatial (ρ)	0.1197** [0.0534]	0.0067 [0.0494]	0.1032* [0.0571]	0.0867 [0.0534]	0.1203** [0.0506]	0.0843* [0.0508]	0.0567 [0.0568]	0.0337 [0.0546]	0.078 [0.0554]
Pseudo R^2^	0.7785	0.8254	0.782	0.7935	0.7959	0.8624	0.859	0.8711	0.8595
**75 km cut-off**									
Coefficient	0.0117 [0.0076]	0.0058* [0.0033]	0.0284** [0.0123]	0.0354*** [0.0109]	0.1978* [0.1171]	0.2444 [0.156]	0.0476 [0.1087]	0.3836** [0.1656]	− 0.0187 [0.0282]
Spatial (ρ)	0.451*** [0.0838]	0.3986*** [0.0872]	0.3882*** [0.0806]	0.4212*** [0.0814]	0.3273*** [0.0724]	0.2837*** [0.0755]	0.1083 [0.073]	0.0938 [0.0677]	0.1167* [0.0691]
Pseudo R^2^	0.8007	0.8406	0.7896	0.813	0.8056	0.8694	0.8592	0.8714	0.8595
**100 km cut-off**									
Coefficient	0.0108 [0.0078]	0.0054 [0.0034]	0.0282** [0.0124]	0.0348*** [0.0112]	0.1919* [0.1124]	0.2434 [0.1621]	0.0294 [0.1096]	0.383** [0.163]	− 0.0058 [0.0291]
Spatial (ρ)	0.4825*** [0.0946]	0.4108*** [0.0999]	0.435*** [0.0924]	0.4429*** [0.0921]	0.4728*** [0.0881]	0.2804*** [0.097]	0.1926* [0.1106]	0.1749* [0.1008]	0.1974* [0.1072]
Pseudo R^2^	0.8058	0.8444	0.7971	0.8173	0.8242	0.8679	0.8628	0.876	0.8626
**No cut-off**									
Coefficient	0.0141** [0.0072]	0.0054* [0.0031]	0.0304** [0.0118]	0.0371*** [0.0104]	0.2116** [0.1072]	0.2602* [0.1568]	0.0128 [0.0912]	0.3181** [0.1446]	0.0044 [0.025]
Spatial (ρ)	0.9135*** [0.0819]	0.8723*** [0.113]	0.901*** [0.0925]	0.9034*** [0.0896]	0.9173*** [0.0777]	0.7917*** [0.1653]	0.8506*** [0.1303]	0.8292*** [0.141]	0.8565*** [0.1266]
Pseudo R^2^	0.8272	0.8633	0.8274	0.8397	0.8554	0.8841	0.8973	0.9055	0.8972

p-value < 0.01***; p-value < 0.05**; p-value < 0.1*. Standard errors in parentheses. All models included a constant and the following controls: dummies for regional capitals and national borders, size of the province, population aged 0–19, distance from nearest airport, share of foreigners, share of male population, population density, degree of urbanization, deaths due to LRT disease, smokers, obese individuals, large firms, altitude, and rainy days.

**Table 12:** Results from SAR models on COVID-19 excess mortality registered on 30 November 2020.

Mortality	NO_2_	O_3_	PM_2.5_	PM_10_	Benzene	BaP	As	Cd	Ni
**50 km cut-off**									
Coefficient	2.0769* [1.1194]	1.952*** [0.5006]	2.3346 [1.8237]	4.1311** [1.7441]	6.3458 [17.888]	− 50.619** [25.16]	40.429** [16.589]	7.9292 [28.04]	− 1.6402 [4.6789]
Spatial (ρ)	0.4526*** [0.0647]	0.4022*** [0.0641]	0.4708 [0.067]	0.4265*** [0.0677]	0.4831*** [0.0646]	0.3312*** [0.0732]	0.1879** [0.0905]	0.2265** [0.0917]	0.2301** [0.0922]
Pseudo R^2^	0.4627	0.5893	0.5056	0.4793	0.4549	0.5932	0.5872	0.531	0.5346
**75 km cut-off**									
Coefficient	1.2783 [0.8548]	1.6234*** [0.4018]	3.5298** [1.4526]	3.3544*** [1.2838]	7.0207 [14.5079]	− 53.418** [22.992]	31.219** [15.356]	7.236 [25.018]	1.8917 [4.1721]
Spatial (ρ)	0.7666*** [0.0561]	0.6862*** [0.0639]	0.6991*** [0.0621]	0.7508*** [0.0576]	0.7228*** [0.0608]	0.5168*** [0.0858]	0.3856*** [0.099]	0.4308*** [0.096]	0.4364*** [0.0964]
Pseudo R^2^	0.5426	0.6481	0.5712	0.5723	0.5396	0.6274	0.6066	0.5469	0.5361
**100 km cut-off**									
Coefficient	1.22 [0.8517]	1.374*** [0.4147]	3.5687** [1.3771]	3.2526** [1.276]	4.5796 [14.451]	− 46.009** [21.952]	29.464** [13.999]	6.4719 [23.031]	4.776 [3.8245]
Spatial (ρ)	0.8354*** [0.0538]	0.7642*** [0.0675]	0.8015*** [0.0581]	0.8219*** [0.0561]	0.806*** [0.0604]	0.6598*** [0.0906]	0.5737*** [0.1063]	0.6111*** [0.1017]	0.6422*** [0.0998]
Pseudo R^2^	0.505	0.6293	0.5416	0.5509	0.5505	0.596	0.5892	0.5183	0.4758
**No cut-off**									
Coefficient	2.5727** [1.1238]	2.3281*** [0.4793]	5.0255*** [1.7987]	5.9237*** [1.6317]	14.632 [18.989]	− 50.39** [25.21]	39.621*** [14.658]	3.6048 [25.536]	2.251 [4.2351]
Spatial (ρ)	0.9495*** [0.0501]	0.9335*** [0.0653]	0.9475*** [0.0521]	0.9483*** [0.0513]	0.9442*** [0.0553]	0.8972*** [0.0999]	0.8847*** [0.1113]	0.8894*** [0.1075]	0.8942*** [0.1035]
Pseudo R^2^	0.5841	0.6753	0.6162	0.6129	0.603	0.6195	0.6701	0.6206	0.6198

p-value < 0.01***; p-value < 0.05**; p-value < 0.1*. Standard errors in parentheses. All models included a constant and the following controls: dummies for regional capitals and national borders, size of the province, population aged 0–19, distance from nearest airport, share of foreigners, share of male population, population density, degree of urbanization, deaths due to LRT disease, smokers, obese individuals, large firms, altitude, and rainy days.

Moreover, since spatially lagged dependent variable ($\rho$) was highly significant in almost all the specifications (Tables 11 and 12), the SAR approach is more appropriate than the classical OLS econometric technique. [Note 14: The use of an ML estimator was also justified by the Cameron and Trivedi’s^116^ decomposition of IM-test over the OLS models, reported in Tables D1 and D2 (Appendix D). All the tests confirmed the hypothesis that OLS errors were homoscedastic and close to a normal distribution, definitively advocating the ML approach [Ref.^117^, p. 236].

Specifically, the outcomes showed that the scalar parameter ρ was large, positive, and verified at a 1% level of significance when a weight matrix with no cut-off is used, suggesting that neighboring provinces tended to display similar patterns in terms of the spread of COVID-19 and excess mortality. [Note 15: Since the scalar parameter ρ always ranged from − 1 to 1 (a sufficient condition for row-standardized weights matrix), the covariance matrix is symmetric positive-definite. Thus, the covariance matrix is correct^132^].

By supposing that other variables remained unchanged, the increase of 1% in the local prevalence resulted in an average increment of 0.87% of COVID-19 prevalence in the adjacent provinces. Similarly, an increase of 1% in the local excess mortality rate resulted in an average increment of 0.92% of excess mortality rate in the neighboring provinces. Notably, when weight matrices with different cut-offs were used (50 km, 75 km, and 100 km), the scalar parameter ρ for excess mortality rate was larger and in some cases more significant than that for prevalence rate.

The high significance of spatially lagged dependent variable may largely be due to the fact that people usually move more easily to neighboring provinces, increasing the likelihood of meeting someone with COVID-19 and spreading the infection.

Moreover, the pseudo R^2^ ranged from 0.78 to 0.91 for COVID-19 prevalence and from 0.45 to 0.68 for excess mortality rate. Since they were significantly higher than 0.2, the models represent an excellent fit [Ref.^133^, p. 35].

For the prevalence rate, NO_2_, PM_2.5_, PM_10_, benzene, and Cd remained positive and statistically significant despite the inclusion of the spillover effect. Notably, the coefficients of Cd increased in statistical significance from 10 to 5% level, while O_3_ switched from 1 to 10% level of significance when a cut-off larger than 50 km was applied (Table 11).

For the excess mortality rate, none of the ambient air pollutants lost its statistical significance. NO_2_, O_3_, PM_2.5_, PM_10_, and As remained positive and highly significant in most cases. BaP increased its level of significance from 10 to 5% (Table 12).

Tables 13 and 14 reported the direct, indirect, and total effect of each air pollutant on prevalence rate and excess mortality rate. The direct effect was almost always significant, while the indirect and total effect were significant especially when weight matrices with different distance cut-offs (50 km, 75 km, and 100 km) were implemented. In other words, air pollutants concentrations in a given province had a significant and positive spillover indirect effect on COVID-19 spread and related mortality in the nearby provinces. For example, as concerns COVID-19 prevalence, PM_10_ had a direct effect ranging from 0.035 and 0.048, an indirect effect ranging from 0.023 to 0.026, and a total effect ranging from 0.052 to 0.062 (Table 13). [Note 16: Only statistically significant coefficients are considered]. Thus, a 1 μg/m^3^ increase in PM_10_ concentrations caused an increment of COVID-19 prevalence ranging from 0.05 to 0.06%. [Note 17: Similarly, for excess mortality, As had a direct effect ranging from 33.4 and 44.9, an indirect effect ranging from 15.1 to 34, and a total effect ranging from 45.3 to 67.8 (Table 14). Consequently, a 1 ng/m^3^ increase in As concentrations caused an increment of excess mortality rate ranging from 45.3 and 67.8 deaths per 100,000 inhabitants]. Generally, the direct effects were greater than spillover effects, suggesting that air pollution concentrations in a province had a larger adverse effect on the same province than in the neighboring provinces. [Note 18: The statistical significance of the spillover indirect effect of air pollutants may also indicate a certain degree of industrial clustering]. Moreover, among common air pollutants, PM_10_ and PM_2.5_ showed the highest total positive effect both for prevalence and excess mortality rate. While, among PAHs and heavy metals, Cd and As showed the total highest effect for prevalence and excess mortality rate, respectively.

**Table 13:** Direct, indirect, and total effects of air pollutants after fitting SAR models on COVID-19 prevalence (on 30 November 2020).

Prevalence	NO_2_	O_3_	PM_2.5_	PM_10_	Benzene	BaP	As	Cd	Ni
**50 km cut-off**									
Direct	0.0242***	0.0112***	0.0352**	0.0489***	0.2563**	0.2194	0.0682	0.393**	− 0.0289
Indirect	0.0024*	0.0005	0.0028*	0.0035	0.0216	0.0115	0.002	0.0069	− 0.0012
Total	0.0266***	0.0113***	0.0379**	0.0523***	0.2779**	0.2309	0.0702	0.3999**	− 0.0301
**75 km cut-off**									
Direct	0.0126	0.0061*	0.0301**	0.0376***	0.2057*	0.2521	0.0478	0.385**	− 0.0188
Indirect	0.0087	0.0035*	0.016**	0.0233***	0.084	0.0811	0.0049	0.0337	− 0.0021
Total	0.0213	0.0096*	0.046**	0.0609***	0.2897*	0.3332	0.0527	0.4187**	− 0.0209
**100 km cut-off**									
Direct	0.0114	0.0056	0.0296**	0.0364***	0.2045*	0.2486	0.0298	0.3866**	− 0.0058
Indirect	0.0095	0.0035*	0.0203**	0.026**	0.1595	0.0871	0.0064	0.0748	− 0.0013
Total	0.0209	0.0092*	0.0499**	0.0624***	0.364*	0.3357	0.0362	0.4615**	− 0.0071
**No cut-off**									
Direct	0.0156**	0.0058*	0.0333**	0.0405***	0.2392*	0.2737*	0.014	0.3448**	0.0049
Indirect	0.1479	0.0367	0.2732	0.3438	2.3178	0.9755	0.0713	1.5178	0.0259
Total	0.1634	0.0426	0.3065	0.3843	2.5571	1.2492	0.0853	1.8627	0.0308

p-value < 0.01***; p-value < 0.05**; p-value < 0.1*.

**Table 14:** Direct, indirect, and total effects of air pollutants after fitting SAR models on COVID-19 related mortality (on 30 November 2020).

Mortality	NO_2_	O_3_	PM_2.5_	PM_10_	Benzene	BaP	As	Cd	Ni
**50 km cut-off**									
Direct	2.3269*	2.1134***	2.6286	4.5615**	7.2183	− 53.23**	40.967**	8.0853	− 1.6735
Indirect	1.0979*	0.7499***	1.2263	1.9812**	3.1039	− 12.499*	4.295	1.0438	− 0.2199
Total	3.4248*	2.8633***	3.8549	6.5428**	10.3222	− 65.73**	45.262**	9.1291	− 1.8934
**75 km cut-off**									
Direct	1.7172	2.0314***	4.5455**	4.4158***	9.2222	− 60.233**	33.386**	7.887	2.0672
Indirect	3.7215	3.1054***	7.0176**	8.9492**	15.269	− 45.629*	15.144*	4.187	1.1182
Total	5.4387	5.1368***	11.5631**	13.365**	24.491	− 105.86**	48.529**	12.074	3.1854
**100 km cut-off**									
Direct	1.6667	1.736***	4.8023***	4.3457***	6.2353	− 54.388**	33.805**	7.6199	5.7642
Indirect	5.747	4.0897***	13.174**	13.92**	17.376	− 78.399*	33.985*	8.6825	7.2964
Total	7.4138	5.8258***	17.977**	18.266**	23.611	− 132.79*	67.79**	16.302	13.061
**No cut-off**									
Direct	3.0365**	2.6732***	5.9807**	6.9644***	17.531	− 56.517*	44.895***	4.1072	2.5805
Indirect	47.926	32.356	89.77	107.51	244.88	− 433.78	298.83	28.476	18.689
Total	50.962	35.029	95.75	114.47	262.41	− 490.302	343.72	32.584	21.27

p-value < 0.01***; p-value < 0.05**; p-value < 0.1*.

Finally, as a further sensitivity check, in Tables 15 and 16, I computed the SAR models for the prevalence and excess mortality rate registered approximately 1 year after the start of the outbreak, that is, on 28 February 2021. [Note 19: The formula used for calculating the excess mortality rate on 28 February 2021 was: $Exces{s}_{mortality}=\mathrm{100,000}\times \left( \frac{{deaths}_{2020-2021}}{{\overline{pop} }_{2020-2021}}- \frac{{\overline{deaths} }_{2015-2019}}{{\overline{pop} }_{2015-2019}}\right)$. Where $deaths_{2020-2021}$ refers to the cumulative deaths from all causes registered from 1 March 2020 to 28 February 2021, ${\overline{deaths} }_{2015-2019}$ is the five-year average deaths (2015—2019) from all causes (from 1 January to 31 December), ${\overline{pop} }_{2020-2021}$ means the average population in the two-year period 2020–2021, and ${\overline{pop} }_{2015-2019}$ is the average population in the 5-year period 2015–2019].

**Table 15:** Results from SAR models on COVID-19 prevalence registered on 28 February 2021.

Prevalence	NO_2_	O_3_	PM_2.5_	PM_10_	Benzene	BaP	As	Cd	Ni
**50 km cut-off**									
Coefficient	0.0353** [0.0139]	0.0105 [0.0066]	0.0704*** [0.0252]	0.0612*** [0.0214]	0.2008 [0.229]	0.844** [0.3434]	0.0513 [0.2312]	0.8236** [0.3754]	− 0.167*** [0.0602]
Spatial (ρ)	0.104** [0.0529]	0.0664 [0.0496]	0.0577 [0.0581]	0.0666 [0.0544]	0.0696 [0.0514]	0.1242** [0.0547]	0.0642 [0.0636]	0.0269 [0.0628]	0.111* [0.0607]
Pseudo R^2^	0.6943	0.7309	0.7099	0.7065	0.7017	0.7534	0.6926	0.7202	0.7224
**75 km cut-off**									
Coefficient	0.0247** [0.0119]	0.0066 [0.0057]	0.0509** [0.0216]	0.0429** [0.0182]	0.1924 [0.2141]	0.7672** [0.3157]	0.0588 [0.234]	0.8426** [0.3659]	− 0.137** [0.0595]
Spatial (ρ)	0.5016*** [0.0787]	0.4443*** [0.0871]	0.3943*** [0.0842]	0.4835*** [0.0804]	0.2731*** [0.079]	0.3386*** [0.0814]	0.0601 [0.0813]	0.0306 [0.0776]	0.064 [0.076]
Pseudo R^2^	0.7181	0.7498	0.7199	0.7332	0.7074	0.7572	0.6907	0.7187	0.7145
**100 km cut-off**									
Coefficient	0.0211* [0.0121]	0.0053 [0.0058]	0.0434** [0.0214]	0.0382** [0.0184]	0.237 [0.1957]	0.6414** [0.3024]	− 0.0479 [0.2137]	0.6931** [0.3398]	− 0.1044* [0.0571]
Spatial (ρ)	0.5793*** [0.0891]	0.5269*** [0.0999]	0.5169*** [0.0974]	0.5607*** [0.0916]	0.5462*** [0.1]	0.4939*** [0.0946]	0.3575*** [0.1148]	0.315*** [0.1128]	0.3128*** [0.114]
Pseudo R^2^	0.7268	0.7573	0.7402	0.7389	0.7393	0.7741	0.6962	0.7255	0.7182
**No cut-off**									
Coefficient	0.0239* [0.0127]	0.0066 [0.0058]	0.057*** [0.0212]	0.0471** [0.0189]	0.2006 [0.2044]	0.79** [0.3206]	0.0317 [0.2057]	0.6773** [0.3308]	− 0.1133** [0.0546]
Spatial (ρ)	0.9079*** [0.0888]	0.8855*** [0.1086]	0.8887*** [0.1063]	0.901*** [0.0949]	0.8895*** [0.1049]	0.8564*** [0.1352]	0.8365*** [0.1529]	0.8105*** [0.173]	0.8212*** [0.1643]
Pseudo R^2^	0.7439	0.7718	0.7537	0.7522	0.7579	0.7797	0.733	0.7524	0.75

p-value < 0.01***; p-value < 0.05**; p-value < 0.1*. Standard errors in parentheses. All models included a constant and the following controls: dummies for regional capitals and national borders, size of the province, population aged 0–19, distance from nearest airport, share of foreigners, share of male population, population density, degree of urbanization, deaths due to LRT disease, smokers, obese individuals, large firms, altitude, and rainy days.

**Table 16:** Results from SAR models on COVID-19 excess mortality registered on 28 February 2021.

Mortality	NO_2_	O_3_	PM_2.5_	PM_10_	Benzene	BaP	As	Cd	Ni
**50 km cut-off**									
Coefficient	2.8423** [1.2349]	2.3784*** [0.5507]	4.1476** [2.0834]	5.6872*** [1.9499]	10.38 [19.832]	− 38.399 [26.303]	50.115*** [16.627]	41.041 [28.385]	− 5.5223 [4.7558]
Spatial (ρ)	0.3673*** [0.0673]	0.2922*** [0.0654]	0.344*** [0.0728]	0.3239*** [0.0715]	0.3846*** [0.0679]	0.3055*** [0.0693]	0.0811 [0.082]	0.1193 [0.0841]	0.1396 [0.0848]
Pseudo R^2^	0.4508	0.5951	0.4968	0.4856	0.4495	0.6148	0.6254	0.5701	0.5682
**75 km cut-off**									
Coefficient	1.8571** [0.939]	1.8935*** [0.452]	4.9649*** [1.6038]	4.4859*** [1.4086]	13.183 [15.512]	− 38.6085 [23.5874]	36.909** [15.436]	38.082 [24.76]	− 2.2329 [4.2121]
Spatial (ρ)	0.7302*** [0.0611]	0.6185*** [0.0725]	0.6337*** [0.0694]	0.7091*** [0.0629]	0.6829*** [0.0654]	0.5075*** [0.0807]	0.3208*** [0.0907]	0.3741*** [0.0871]	0.3724*** [0.089]
Pseudo R^2^	0.5398	0.6508	0.5584	0.5762	0.5343	0.655	0.6399	0.5834	0.5791
**100 km cut-off**									
Coefficient	1.7917* [0.9222]	1.658*** [0.4583]	4.9938*** [1.4777]	4.302*** [1.381]	15.061 [15.806]	− 39.121* [23.595]	36.757** [14.264]	37.408 [23.304]	1.263 [4.087]
Spatial (ρ)	0.8139*** [0.0587]	0.7151*** [0.0777]	0.7696*** [0.0647]	0.796*** [0.0615]	0.7651*** [0.0695]	0.6154*** [0.0943]	0.5005*** [0.108]	0.5497*** [0.1033]	0.5613*** [0.1076]
Pseudo R^2^	0.5236	0.6454	0.5451	0.5747	0.5543	0.5888	0.6332	0.5569	0.5264
**No cut-off**									
Coefficient	2.8712** [1.1623]	2.4814*** [0.4884]	6.2957*** [1.8227]	6.7678*** [1.6727]	19.24 [19.078]	− 36.032 [26.203]	46.58*** [14.277]	37.734 [25.042]	− 1.6729 [4.2456]
Spatial (ρ)	0.9495*** [0.0502]	0.9313*** [0.0675]	0.9436*** [0.0559]	0.9479*** [0.0517]	0.9441*** [0.0554]	0.8979*** [0.0993]	0.8791*** [0.116]	0.8807*** [0.1151]	0.878*** [0.118]
Pseudo R^2^	0.5882	0.6845	0.6242	0.6264	0.6143	0.6327	0.7009	0.6426	0.6346

p-value < 0.01***; p-value < 0.05**; p-value < 0.1*. Standard errors in parentheses. All models included a constant and the following controls: dummies for regional capitals and national borders, size of the province, population aged 0–19, distance from nearest airport, share of foreigners, share of male population, population density, degree of urbanization, deaths due to LRT disease, smokers, obese individuals, large firms, altitude, and rainy days.

The results confirmed the statistical significance of the spatially lagged dependent variable (ρ), that was almost always large and positive. Moreover, outdoor air pollutants substantially maintained a high statistical significance, although the latter had changed in some cases. Regarding to COVID-19 prevalence, benzene became not significant at all, and BaP increased in statistical significance from 5 to 1% (Table 15). [Note 20: Notably, the coefficient of Ni becomes statistically significant, even if the association with COVID-19 prevalence was negative]. Table 16 showed that just the impact of BaP on excess mortality was not confirmed. In fact, its coefficient, although still negative, became not significant.

Thus, the results are robust to changes in the specifications and show the persistence of the link between environmental pollution and the transmission and mortality of COVID-19, also suggesting the potentially dangerous effect of PAHs and heavy metals, such as benzene, BaP, As, and Cd.

## Limitations

This study has three main limitations: (1) first, the sample size is not large, ranging from 60 to 107 observations, that is the Italian provinces; (2) since pollution monitors are sparsely located in some specific provincials’ areas, such as specific traffic and industrial provincial capitals’ areas, the study may suffer from exposure measurement errors, that is the discrepancy between outdoor air pollutants concentration and personal air pollution exposure; (3) notwithstanding the study considers a wide range of potential covariates, it is not possible to grasp and include all the aspects that may affect COVID-19 spread and related mortality.

## Conclusions

In this article, I investigated the common sources of outdoor air pollution and the global air quality in the 107 Italian provinces in the period 2014–2019, and the link between long-term exposure to nine air pollutants in the same period and COVID-19 spread and related mortality. The major strengths of this study are the implementation of nine air pollutants, 18 potential covariates, and three different statistical methodologies (NB, OLS, and SAR) to address the robustness of the associations.

The results showed that: (i) common air pollutants (NO_2_, O_3_, PM_2.5_, and PM_10_) and PAHs (benzene and BaP) exhibited a positive and significant correlation with the presence of large firms, energy and gas consumption, vehicles density, public transport, cattle fodder, and livestock density; (ii) the provinces located in the north of Italy were generally much more polluted than the southern ones; (iii) long-term exposure to NO_2_, PM_2.5_, and PM_10_, benzene, BaP, and Cd was positively correlated with the spread of COVID-19 infections across the Italian provinces; and (iv) long-term exposure to NO_2_, O_3_, PM_2.5_, PM_10_, and As was positively associated with excess mortality due to COVID-19.

The dangerous effect of the common air pollutants NO_2_, O_3_, PM_2.5_ and PM_10_ was consistent with recent literature^11,13,14,17,19,66,67,72^. Moreover, this study found that as well as the common air pollutants, PAHs and heavy metals may also have played a key role in explaining the variability of COVID-19 spread and related mortality. This outcome seems interesting and of relevance, given that these air pollutants have not been considered at all by recent scientific literature. Finally, the results suggest the need for national strategies and economic policies that aim at reducing air pollutant concentrations to improve air quality levels (especially in Northern Italy) and to cope more effectively with similar unexpected pandemics in the future.

## Supplementary Information

Supplementary Information.

## Data Availability

The data used to support the findings of this study are included within the article and the supplementary material. Raw data that support the findings of this study are available from the author, G. P., upon reasonable request.

## References

[1] Worldometer. COVID-19 Coronavirus Pandemic dataset. (2021). https://www.worldometers.info/coronavirus/. Accessed 2 Nov 2021.

[2] World Health Organization (WHO). Tracking SARS-COV-2 variants. (2021). https://www.who.int/en/activities/tracking-SARS-CoV-2-variants/. Accessed 10 May 2021.

[3] World Health Organization (WHO). Ambient air pollution: A global assessment of exposure and burden of disease. World Health Organization. (2016). https://apps.who.int/iris/handle/10665/250141. Accessed 20 May 2021.

[4] European Environmental Agency (EEA). Healthy environment, healthy lives: How the environment influences health and well-being in Europe. (2019). https://www.eea.europa.eu/publications/healthy-environment-healthy-lives. Accessed 20 May 2021.

[5] Schraufnagel DE (2019). Air pollution and noncommunicable diseases: A review by the Forum of International Respiratory Societies’ Environmental Committee, Part 2: Air pollution and organ systems. Chest.

[6] European Environmental Agency (EEA). Air quality in Europe—2020 report, EEA report NO. 09/2020. (2020). https://www.eea.europa.eu/publications/air-quality-in-europe-2020-report. Accessed 20 May 2021.

[7] World Health Organization (WHO). Air quality guidelines global update 2005: Particulate matter, ozone, nitrogen dioxide and sulfur dioxide. (WHO Regional Office for Europe, 2006). https://apps.who.int/iris/handle/10665/107823. Accessed 21 May 2021.

[8] Bashir MF, Bilal BM, Komal B (2020). Correlation between environmental pollution indicators and COVID-19 pandemic: A brief study in Californian context. Environ. Res..

[9] Filippini T, Rothman KJ, Goffi A, Ferrari F, Maffeis G, Orsini N, Vinceti M (2020). Satellite-detected tropospheric nitrogen dioxide and spread of SARS-CoV-2 infection in Northern Italy. Sci. Total Environ..

[10] Zhang Z, Xue T, Jin X (2020). Effects of meteorological conditions and air pollution on COVID-19 transmission: Evidence from 219 Chinese cities. Sci. Total Environ..

[11] Zhu Y, Xie J, Huang F, Cao L (2020). Association between short-term exposure to air pollution and COVID-19 infection: Evidence from China. Sci. Total Environ..

[12] De Angelis E, Renzetti S, Volta M, Donato F, Calza S, Placidi D (2021). COVID-19 incidence and mortality in Lombardy, Italy: An ecological study on the role of air pollution, meteorological factors, demographic and socioeconomic variables. Environ. Res..

[13] Solimini A, Filipponi F, Fegatelli DA, Caputo B, De Marco CM, Spagnoli A, Vestri AR (2021). A global association between Covid-19 cases and airborne particulate matter at regional level. Sci. Rep..

[14] Cole MA, Ozgen C, Strobl E (2020). Air pollution exposure and Covid-19 in Dutch municipalities. Environ. Resour. Econ..

[15] Hendryx M, Luo J (2020). COVID-19 prevalence and fatality rates in association with air pollution emission concentrations and emission sources. Environ. Pollut..

[16] Pozzer A (2020). Regional and global contributions of air pollution to risk of death from COVID-19. Cardiovasc. Res..

[17] Perone G (2021). The determinants of COVID-19 case fatality rate (CFR) in the Italian regions and provinces: An analysis of environmental, demographic, and healthcare factors. Sci. Total Environ..

[18] Travaglio M, Yu Y, Popovic R, Selley L, Leal NS, Martins LM (2021). Links between air pollution and COVID-19 in England. Environ. Pollut..

[19] Becchetti L, Conzo G, Conzo P, Salustri F (2022). Understanding the heterogeneity of COVID-19 deaths and contagions: The role of air pollution and lockdown decisions. J. Environ. Manag..

[20] Bontempi E (2020). First data analysis about possible COVID-19 virus airborne diffusion due to air particulate matter (PM): The case of Lombardy (Italy). Environ. Res..

[21] Coccia M (2020). Factors determining the diffusion of COVID-19 and suggested strategy to prevent future accelerated viral infectivity similar to COVID. Sci. Total Environ..

[22] Comunian S, Dongo D, Milani C, Palestini P (2020). Air pollution and COVID-19: The role of particulate matter in the spread and increase of COVID-19’s morbidity and mortality. Int. J. Environ. Res. Public Health.

[23] Fattorini D, Regoli F (2020). Role of the chronic air pollution levels in the Covid-19 outbreak risk in Italy. Environ. Pollut..

[24] Lolli S, Chen YC, Wang SH, Vivone G (2020). Impact of meteorological conditions and air pollution on COVID-19 pandemic transmission in Italy. Sci. Rep..

[25] Zoran MA, Savastru RS, Savastru DM, Tautan MN (2020). Assessing the relationship between surface levels of PM2.5 and PM10 particulate matter impact on COVID-19 in Milan, Italy. Sci. Total Environ..

[26] Collivignarelli MC (2021). Can particulate matter be identified as the primary cause of the rapid spread of CoViD-19 in some areas of Northern Italy?. Environ. Sci. Pollut. Res..

[27] Kaxiras E, Neofotistos G (2020). Multiple epidemic wave model of the COVID-19 pandemic: Modeling study. J. Med. Internet Res..

[28] Perone, G. Comparison of ARIMA, ETS, NNAR, TBATS and hybrid models to forecast the second wave of COVID-19 hospitalizations in Italy. *Eur. J. Health Econ.* 1–24 (2021). 10.1007/s10198-021-01347-4 PMC8332000 34347175

[29] Perone G (2022). Using the SARIMA model to forecast the fourth global wave of cumulative deaths from COVID-19: Evidence from 12 hard-hit big countries. Econometrics.

[30] European Environmental Agency (EEA). Air pollution sources. (2020). https://www.eea.europa.eu/themes/air/air-pollution-sources-1. Accessed 10 June 2021.

[31] European Environmental Agency (EEA). Air quality in Europe—2016 report. No. 28/2016. (2016). https://www.eea.europa.eu/publications/air-quality-in-europe-2016. Accessed 10 June 2021.

[32] De Donno A, De Giorgi M, Bagordo F, Grassi T, Idolo A, Serio F (2018). Health risk associated with exposure to PM10 and benzene in three Italian towns. Int. J. Environ. Res. Public Health.

[33] Lovarelli D, Conti C, Finzi A, Bacenetti J, Guarino M (2020). Describing the trend of ammonia, particulate matter and nitrogen oxides: The role of livestock activities in northern Italy during Covid-19 quarantine. Environ. Res..

[34] World Health Organization (WHO). Preventing Disease Through Healthy Environments. Exposure to benzene: a major public health concern. (2010). https://www.who.int/ipcs/features/benzene.pdf. Accessed 10 June 2021.

[35] United States Environmental Protection Agency (US EPA 2017). Toxicological Review of Benzo[a]pyrene (EPA/635/R-17/003Fc). Washington DC: US. https://iris.epa.gov/static/pdfs/0136_summary.pdf. Accessed 10 June 2021.

[36] Italian National Institute of Statistics (ISTAT). I.Stat database. (2021). http://dati.istat.it. Accessed 20 July 2021.

[37] Italian National Institute of Statistics (ISTAT). Principali fattori di pressione sull’ambiente nelle città italiane. (2021). https://www.istat.it/it/archivio/252928. Accessed 23 July 2021.

[38] Italian National Institute of Statistics (ISTAT). Ambiente Urbano. (2021). https://www.istat.it/it/archivio/254037. Accessed 25 July 2021.

[39] Italian National Institute of Statistics (ISTAT). Ambiente Urbano. (2021). https://www.istat.it/it/archivio/258691. Accessed 28 July 2021.

[40] Italian National Institute of Statistics (ISTAT). Ambiente Urbano. (2017). https://www.istat.it/it/archivio/207482. Accessed 3 Aug 2021.

[41] Webb J (2005). Managing ammonia emissions from livestock production in Europe. Environ. Pollut..

[42] Laubach J (2013). Ammonia emissions from cattle urine and dung excreted on pasture. Biogeosciences.

[43] McCubbin DR, Apelberg BJ, Roe S, Divita F (2002). Livestock ammonia management and particulate-related health benefits. Environ. Sci. Technol..

[44] Greenpace-ISPRA. Covid-19, esposizione al particolato e allevamenti intensivi. (2020). https://www.greenpeace.org/static/planet4-italy-stateless/2020/04/184484ca-ricerca-particolato-def.pdf. Accessed 10 Aug 2021.

[45] European Commission. Air Quality Standards. https://ec.europa.eu/environment/air/quality/standards.htm. Accessed 24 Mar 2021.

[46] World Health Organization (WHO). WHO Expert Consultation: Available evidence for the future update of the WHO Global Air Quality Guidelines (AQGs). (2015). https://www.euro.who.int/__data/assets/pdf_file/0013/301720/Evidence-future-update-AQGs-mtg-report-Bonn-sept-oct-15.pdf. Accessed 28 Apr 2021.

[47] European Environmental Agency (EEA). European city air quality viewer. January 17, 2021. (2021). https://www.eea.europa.eu/themes/air/urban-air-quality/european-city-air-quality-viewer. Accessed 29 Apr 2021.

[48] Malanima P, Zamagni V (2010). 150 years of the Italian economy, 1861–2010. J. Mod. Ital. Stud..

[49] Bigoni M, Bortolotti S, Casari M, Gambetta D (2019). At the root of the North-South cooperation gap in Italy: Preferences or beliefs?. Econ. J..

[50] Cadelis G, Tourres R, Molinie J (2014). Short-term effects of the particulate pollutants contained in Saharan dust on the visits of children to the emergency department due to asthmatic conditions in Guadeloupe (French Archipelago of the Caribbean). PLoS One.

[51] Goeminne PC (2018). The impact of acute air pollution fluctuations on bronchiectasis pulmonary exacerbation: A case-crossover analysis. Eur. Respir. J..

[52] Liang L (2019). Associations between daily air quality and hospitalisations for acute exacerbation of chronic obstructive pulmonary disease in Beijing, 2013–17: An ecological analysis. Lancet Planet. Health.

[53] Murdoch DR, Jennings LC (2009). Association of respiratory virus activity and environmental factors with the incidence of invasive pneumococcal disease. J. Infect..

[54] Xing DF (2019). Spatial association between outdoor air pollution and lung cancer incidence in China. BMC Public Health.

[55] Zeng Q (2017). The association between ambient inhalable particulate matter and the disease burden of respiratory disease: An ecological study based on ten-year time series data in Tianjin, China. Environ. Res..

[56] Låg M, Øvrevik J, Refsnes M, Holme JA (2020). Potential role of polycyclic aromatic hydrocarbons in air pollution-induced non-malignant respiratory diseases. Respir. Res..

[57] Nemery B (1990). Metal toxicity and the respiratory tract. Eur. Respir. J..

[58] Gu Q, Lin RL (2010). Heavy metals zinc, cadmium, and copper stimulate pulmonary sensory neurons via direct activation of TRPA1. J. Appl. Physiol..

[59] Ahmed S (2017). Arsenic exposure alters lung function and airway inflammation in children: A cohort study in rural Bangladesh. Environ. Int..

[60] Mo Y (2019). Comparative mouse lung injury by nickel nanoparticles with differential surface modification. J. Nanobiotechnol..

[61] Hays AM, Srinivasan D, Witten ML, Carter DE, Lantz RC (2006). Arsenic and cigarette smoke synergistically increase DNA oxidation in the lung. Toxicol. Pathol..

[62] Rokadia HK, Agarwal S (2013). Serum heavy metals and obstructive lung disease: Results from the National Health and Nutrition Examination Survey. Chest.

[63] Klein EF (2021). Trajectory of inhaled cadmium ultrafine particles in smokers. BMJ Open Respir. Res..

[64] Xiao T (2021). LncRNA H19-mediated M2 polarization of macrophages promotes myofibroblast differentiation in pulmonary fibrosis induced by arsenic exposure. Environ. Pollut..

[65] Liang D (2020). Urban air pollution may enhance COVID-19 case-fatality and mortality rates in the United States. Innovation.

[66] Dales R (2021). The association between air pollution and COVID-19 related mortality in Santiago, Chile: A daily time series analysis. Environ. Res..

[67] Bolaño-Ortiz TR (2020). Spread of SARS-CoV-2 through Latin America and the Caribbean region: A look from its economic conditions, climate and air pollution indicators. Environ. Res..

[68] Delnevo G, Mirri S, Roccetti M (2020). Particulate matter and COVID-19 disease diffusion in Emilia-Romagna (Italy). Already a cold case?. Computation.

[69] Hutter HP (2020). Air pollution is associated with COVID-19 incidence and mortality in Vienna, Austria. Int. J. Environ. Res. Public Health.

[70] Li H (2020). Air pollution and temperature are associated with increased COVID-19 incidence: A time series study. Int. J. Infect. Dis..

[71] Lin S (2020). Region-specific air pollutants and meteorological parameters influence COVID-19: A study from mainland China. Ecotoxicol. Environ. Saf..

[72] Vasquez-Apestegui BV (2021). Association between air pollution in Lima and the high incidence of COVID-19: Findings from a post hoc analysis. BMC Public Health.

[73] López-Feldman A, Heres D, Marquez-Padilla F (2021). Air pollution exposure and COVID-19: A look at mortality in Mexico City using individual-level data. Sci. Total Environ..

[74] Berry, W. D., & Feldman, S. *Multiple Regression in Practice*, vol. 50. (SAGE Publications, 1985).

[75] Donath C (2012). Predictors of binge drinking in adolescents: Ultimate and distal factors-a representative study. BMC Public Health.

[76] Ahmed A, Ali A, Hasan S (2020). Comparison of epidemiological variations in COVID-19 patients inside and outside of China—A meta-analysis. Front. Public Health.

[77] Forsblom E (2021). Male predominance in disease severity and mortality in a low Covid-19 epidemic and low case-fatality area—A population-based registry study. Infect. Dis..

[78] Nguyen NT (2021). Male gender is a predictor of higher mortality in hospitalized adults with COVID-19. PLoS One.

[79] Chan JFW (2020). A familial cluster of pneumonia associated with the 2019 novel coronavirus indicating person-to-person transmission: A study of a family cluster. Lancet.

[80] Poletti P (2021). Association of age with likelihood of developing symptoms and critical disease among close contacts exposed to patients with confirmed SARS-CoV-2 infection in Italy. JAMA Netw. Open.

[81] Sridhar KS (2021). Urbanization and COVID-19 prevalence in India. Reg. Sci. Policy Pract..

[82] Martins-Filho PR (2021). Relationship between population density and COVID-19 incidence and mortality estimates: A county-level analysis. J. Infect. Public Health.

[83] Bhadra A, Mukherjee A, Sarkar K (2021). Impact of population density on Covid-19 infected and mortality rate in India. Model. Earth Syst. Environ..

[84] Ilardi, A., Chieffi, S., Iavarone, A., & Ilardi, C. R. (2020). SARS-CoV-2 in Italy: Population density correlates with morbidity and mortality. Jpn. J. Infect. Dis. JJID-2020. 10.7883/yoken.JJID.2020.200 32611978

[85] Jordan RE, Adab P, Cheng K (2020). Covid-19: Risk factors for severe disease and death. BMJ.

[86] Istat-ISS 2020. Impatto dell'epidemia COVID-19 sulla mortalità totale della popolazione residente primo trimestre 2020. May 4, 2020. https://www.istat.it/it/files/2020/05/Rapporto_Istat_ISS.pdf. Accessed 3 May 2022.

[87] Alkhathami MG (2021). Prevalence and mortality of lung comorbidities among patients with COVID-19: A systematic review and meta-analysis. Lung India.

[88] Gülsen A, König IR, Jappe U, Drömann D (2021). Effect of comorbid pulmonary disease on the severity of COVID-19: A systematic review and meta-analysis. Respirology.

[89] Giacomelli A (2020). 30-day mortality in patients hospitalized with COVID-19 during the first wave of the Italian epidemic: A prospective cohort study. Pharmacol. Res..

[90] Docherty AB (2020). Features of 20 133 UK patients in hospital with covid-19 using the ISARIC WHO Clinical Characterisation Protocol: Prospective observational cohort study. BMJ.

[91] Klang E (2020). Severe obesity as an independent risk factor for COVID-19 mortality in hospitalized patients younger than 50. Obesity.

[92] Lippi G, Henry BM (2020). Active smoking is not associated with severity of coronavirus disease 2019 (COVID-19). Eur. J. Intern. Med..

[93] Usman MS (2021). Is there a smoker’s paradox in COVID-19?. BMJ Evid. Based Med..

[94] Huang C (2020). Clinical features of patients infected with 2019 novel coronavirus in Wuhan, China. Lancet.

[95] Wenzl, T. Smoking and COVID-19—A review of studies which motivated unexpected health claims, EUR 30373 EN, Publications Office of the European Union, Luxembourg, 2020, ISBN 978-92-76-22062-6. 10.2760/564217, JRC121837. Accessed 7 May 2022.

[96] Meini S, Fortini A, Andreini R, Sechi LA, Tascini C (2021). The paradox of the low prevalence of current smokers among COVID-19 patients hospitalized in nonintensive care wards: Results from an Italian multicenter case-control study. Nicotine Tob. Res..

[97] Cano-Pérez E (2020). Negative correlation between altitude and COVID-19 pandemic in Colombia: A preliminary report. Am. J. Trop. Med. Hyg..

[98] Accinelli RA, Leon-Abarca JA (2020). En la altura la COVID-19 es menos frecuente: la experiencia del Perú. Arch. Bronconeumol..

[99] Stephens KE, Chernyavskiy P, Bruns DR (2021). Impact of altitude on COVID-19 infection and death in the United States: A modeling and observational study. PLoS One.

[100] Huamaní C, Velásquez L, Montes S, Miranda-Solis F (2020). Propagation by COVID-19 at high altitude: Cusco case. Respir. Physiol. Neurobiol..

[101] Tosepu R (2020). Correlation between weather and Covid-19 pandemic in Jakarta, Indonesia. Sci. Total Environ..

[102] Liu J (2020). Impact of meteorological factors on the COVID-19 transmission: A multi-city study in China. Sci. Total Environ..

[103] Wu Y (2020). Effects of temperature and humidity on the daily new cases and new deaths of COVID-19 in 166 countries. Sci. Total Environ..

[104] Christophi CA (2021). Ambient temperature and subsequent COVID-19 mortality in the OECD countries and individual United States. Sci. Rep..

[105] Tapia-Muñoz T (2022). COVID-19 attributed mortality and ambient temperature: A global ecological study using a two-stage regression model. Pathog. Glob. Health.

[106] Majumder P, Ray PP (2021). A systematic review and meta-analysis on correlation of weather with COVID-19. Sci. Rep..

[107] Shenoy A (2022). God is in the rain: The impact of rainfall-induced early social distancing on COVID-19 outbreaks. J. Health Econ..

[108] Italian National Institute of Statistics (ISTAT). Decessi e cause di morte: cosa produce l’ISTAT. (2022). https://www.istat.it/it/archivio/240401. Accessed 18 Apr 2022.

[109] Italian Ministry of Health. COVID-19, Dati Province. (2020). https://github.com/pcm-dpc/COVID-19/tree/master/dati-province. Accessed 5 Apr 2021.

[110] Sole 24 Ore. Lab24: Tamponi giornalieri e contagiati. (2021). https://lab24.ilsole24ore.com/coronavirus/. Accessed 15 Jan 2021.

[111] Mathieu E (2021). A global database of COVID-19 vaccinations. Nat. Hum. Behav..

[112] Cameron AC, Johansson P (1997). Count data regression using series expansions: With applications. J. Appl. Economet..

[113] Anselin L (1988). Spatial Econometrics: Methods and Models.

[114] Moran PA (1948). The interpretation of statistical maps. J. R. Stat. Soc. Ser. B (Methodol.).

[115] Cliff AD, Ord JK (1973). Spatial Autocorrelation.

[116] Cameron, A. C., & Trivedi, P. K. The information matrix test and its applied alternative hypotheses. In *Working paper No. 372, University of California–Davis, Institute of Governmental Affairs* (1990).

[117] Drukker DM, Prucha IR, Raciborski R (2013). Maximum likelihood and generalized spatial two-stage least-squares estimators for a spatial-autoregressive model with spatial-autoregressive disturbances. Stand. Genom. Sci..

[118] McFadden, D. Conditional logit analysis of qualitative choice behavior. In *Frontiers in Econometrics* (ed. Zarembka, P.) 105–142 (Academic Press, 1974). https://eml.berkeley.edu/reprints/mcfadden/zarembka.pdf. Accessed 12 Sep 2021.

[119] MacKinnon JG, White H (1985). Some heteroskedasticity-consistent covariance matrix estimators with improved finite sample properties. J. Econom..

[120] Davidson R, MacKinnon JG (1993). Estimation and Inference in Econometrics.

[121] Rogerson PA (2001). Statistical Methods for Geography.

[122] European Commission. Annual report on intra-EU labour mobility 2020. (2021). https://op.europa.eu/en/publication-detail/-/publication/ab706f9b-74bf-11eb-9ac9-01aa75ed71a1/language-en. Accessed 4 May 2022.

[123] Wong DW, Li Y (2020). Spreading of COVID-19: Density matters. PLoS One.

[124] Diao Y (2021). Influence of population density, temperature, and absolute humidity on spread and decay durations of COVID-19: A comparative study of scenarios in China, England, Germany, and Japan. One Health.

[125] Segovia-Juarez J, Castagnetto JM, Gonzales GF (2020). High altitude reduces infection rate of COVID-19 but not case-fatality rate. Respir. Physiol. Neurobiol..

[126] Arias-Reyes C (2021). Decreased incidence, virus transmission capacity, and severity of COVID-19 at altitude on the American continent. PLoS One.

[127] Fernandes JSC (2021). Altitude conditions seem to determine the evolution of COVID-19 in Brazil. Sci. Rep..

[128] Ejaz H (2020). COVID-19 and comorbidities: Deleterious impact on infected patients. J. Infect. Public Health.

[129] Sarkodie SA, Owusu PA (2020). Impact of meteorological factors on COVID-19 pandemic: Evidence from top 20 countries with confirmed cases. Environ. Res..

[130] Tobías A, Molina T (2020). Is temperature reducing the transmission of COVID-19?. Environ. Res..

[131] Chen S (2021). Climate and the spread of COVID-19. Sci. Rep..

[132] LeSage, J., & Pace, R. K. *Introduction to Spatial Econometrics*. (Chapman and Hall/CRC, 2009).

[133] McFadden, D. Quantitative Methods for Analyzing Behaviour of Individuals: Some Recent Developments, Cowles Fundation Discussion Paper No. 474, Yale University. (1977). https://cowles.yale.edu/sites/default/files/files/pub/d04/d0474.pdf. Accessed 5 June 2022.

