# Supplementary material for: Assessing the impact of long-term exposure to nine outdoor air pollutants on COVID-19 spatial spread and related mortality in 107 Italian provinces
Source: Sci Rep. 2022 Aug 3;12:13317. doi: 10.1038/s41598-022-17215-x (PMC9349267; doi:10.1038/s41598-022-17215-x)
Supplement: Supplementary file 1 — Supplementary Information. [file 41598_2022_17215_MOESM1_ESM.pdf]

APPENDIX A

Table A1. Correlation matrix: pairwise correlation coefficients between main control variables.

	Aged 0–19	Airp. dist.	Foreigners	Male	Pop. density	Urbanization	LRT disease
Aged 0–19	1						
Airp. dist.	-0.2017	1					
Foreigners	-0.0197	-0.1715	1				
Male	0.3364	0.2112	-0.0525	1			
Pop. density	0.288	-0.383	0.153	-0.1614	1		
Urbanization	0.2663	-0.4931	0.1941	-0.3146	0.5429	1	
LRT disease	-0.3877	0.1798	-0.256	-0.3791	-0.198	-0.1359	1
Smokers	-0.0656	0.016	-0.1048	-0.2355	-0.0712	0.0703	0.2731
Obese	0.2574	0.008	-0.5131	0.0505	-0.1029	-0.1662	0.198
Large firms	0.0626	-0.2818	0.6102	-0.1867	0.371	0.3187	-0.2073
Altitude	-0.1316	0.3949	-0.2349	0.1249	-0.1838	-0.1592	0.1686
Rainy days	-0.0904	0.0841	0.0989	0.1466	0.0947	0.01	-0.0918
Temperature	0.0803	-0.2242	-0.3354	-0.0858	0.0739	0.0133	0.1742

	Smokers	Obese	Large firms	Altitude	Rainy days	Temperature
Smokers	1					
Obese	0.3388	1				
Large firms	-0.0847	-0.3968	1			
Altitude	-0.069	-0.0897	-0.1754	1		
Rainy days	-0.2326	-0.2279	0.1643	0.4337	1	
Temperature	0.278	0.5233	-0.423	-0.4885	-0.4712	1

Notes: correlations > 0.5 (in absolute value) are underlined.

Table A2. Pairwise correlation coefficients between main control variables and air pollutants.

	NO_2	$O_{3\ (>120)}$	$PM_{2.5}$	PM_{10}	Benzene	BaP	As	Cd	Ni
Aged 0–19	0.2177	-0.0008	0.0791	0.2386	0.0444	0.1207	0.2333	0.1032	0.1457
Airp. dist.	-0.2929	-0.1604	-0.1918	-0.318	-0.1664	0.1764	0.1237	0.0729	0.283
Foreigners	0.4387	0.6211	0.5517	0.4256	0.084	0.2265	0.0833	0.1057	0.0417
Male	-0.1343	0.0534	0.0324	0.0683	-0.2947	0.1237	0.2032	-0.0404	0.046
Pop. density	0.4788	0.2211	0.2955	0.3079	0.3446	-0.1127	0.2464	0.118	0.0856
Urbanization	0.4609	0.2064	0.2202	0.2711	0.4319	-0.0587	0.1316	0.1656	0.1251
LRT disease	-0.3361	-0.2158	-0.3093	-0.3289	0.0388	-0.3162	-0.3402	-0.2278	-0.0554
Smokers	-0.1208	-0.3178	-0.2678	-0.0632	0.1951	-0.2608	-0.1352	-0.1113	-0.0608
Obese	-0.3525	-0.4951	-0.3705	-0.1555	-0.0955	-0.3792	-0.3747	-0.2315	-0.191
Large firms	0.568	0.57	0.5675	0.4594	0.2685	0.2118	0.0759	0.0457	-0.0387
Altitude	-0.1872	-0.0325	-0.2706	-0.474	-0.1856	0.2	0.0798	-0.074	0.3093
Rainy days	0.1411	0.2848	0.0555	-0.1037	-0.1868	0.2148	0.1082	-0.0207	-0.0191
Temperature	-0.319	-0.4693	-0.3124	-0.0523	-0.0312	-0.5733	-0.198	-0.0695	-0.2888

Notes: correlations > 0.5 (in absolute value) are underlined.

APPENDIX B

Table B1. Definition of variables used in the empirical analysis.

**Variables**	**Definitions**	**Sources**
Dependent variables		
Confirmed cases	The number of COVID-19 cumulative cases in each province, on 30 November 2020.	Italian Ministry of Health (2020)
Prevalence rate	The ratio between people who have been tested positive for COVID-19 on 30 November 2020 and total resident population on 1 January 2020.	I.Stat (2021a), Italian Ministry of Health (2020)
Excess deaths	The difference between the cumulative number of deaths from all causes from 1 March 2020 to 30 November 2020 and the cumulative number of deaths from all causes from 1 March to 30 November, averaged in the period 2015–2019.	Istat (2022)
Excess mortality	The cumulative excess deaths from 1 March 2020 to 30 November 2020 per 100,000 inhabitants.[1]	I.Stat (2021a), Istat (2022)
Independent variables		
AUT border	A dummy that takes 1 when the province borders Austria and 0 elsewhere.	Google Maps
FRA border	A dummy that takes 1 when the province borders France and 0 elsewhere.	Google Maps
SLO border	A dummy that takes 1 when the province borders Slovenia and 0 elsewhere.	Google Maps
SWI border	A dummy that takes 1 when the province borders Switzerland and 0 elsewhere.	Google Maps
Airport distance	The distance in kilometers between the provincial capital's center and the nearest airport with at least 50,000 passengers over the period January 2020–November 2020.	Assoaeroporti (2021), www.michelin.it
Foreigners	The foreign-born population measured as a percentage of the total resident population in each province, on 1 January 2020.	I.Stat (2021a)
Aged 0–19	The percentage of the resident population aged 0–19 in each province, on 1 January 2020.	I.Stat (2021a)
Male	The percentage of resident population that is male on 1 January 2020.	I.Stat (2021a)
Pop. density	The number of inhabitants per square kilometer of land area in each province, on 1 January 2020.[2]	I.Stat (2021a)
Urbanization	An ordinal index that ranks population of each province by urban-rural structure: predominantly rural (1), intermediate (2), and predominantly urban (3).	Eurostat (2013)
Obesity	The average percentage of obese individuals in each region, in period 2016–2019.	ISS (2021a)
Smokers	The average percentage of smokers in each region, in the period 2016–2019.	ISS (2021b)
LRT disease	The average deaths from chronic lower respiratory tract disease (per 100,000 inhabitants) in each province, in the period 2014–2019.	I.Stat (2021a)
Large firms	The number of firms that employed 250 or more employees (per 100,000 inhabitants) in each province, in the period 2014–2019.	I.Stat (2021a)
Capitals	A dummy that takes 1 when the province is also the regional capital and 0 elsewhere.	I.Stat (2021a)
Province size	The total area of each province expressed in square kilometers.	I.Stat (2021a)
Altitude	The average altitude of the provincial capital.	Istat (2021b)
Rainy days	The average annual days of rain in the provincial capital, in the period 2007–2018	Istat (2020)
Temperature	The average annual temperature of provincial capital, in the period 2009–2018.	Mipaaf (2021)
NO_2	The average concentrations of nitrogen dioxide, expressed in micrograms per cubic meter of air ($\mu g/m^3$), in the period 2014–2019.[3]	Istat (2015, 2017, 2019), Legambiente (2020)
$O_{3\ (>120)}$	The average number of days in which ozone exceeded the limit of 120 micrograms per cubic meter of air ($\mu g/m^3$), in the period 2014–2019.	Istat (2015, 2017 2019), Legambiente (2020)
$O_{3\ (>180)}$	The average number of hours in which ozone exceeded the limit of 180 micrograms per cubic meter of air ($\mu g/m^3$) in each province, in the period 2014–2018.	Istat (2015, 2017, 2019)
$PM_{2.5}$	The average concentrations of particulate matter less than 2.5 micrometers in diameter, expressed in micrograms per cubic meter of air ($\mu g/m^3$), in the period 2014–2019.	Istat (2015, 2017, 2019), Legambiente (2020)
PM_{10}	The average concentrations of particulate matter less than 10 micrometers in diameter, expressed in micrograms per cubic meter of air ($\mu g/m^3$), in the period 2014–2019.	Istat (2015, 2017, 2019), Legambiente (2020)
$PM_{10\ (>50)}$	The average number of days in which PM_{10} exceeded the limit of 50 micrograms per cubic meter of air ($\mu g/m^3$), in the period 2014–2018.	Istat (2015, 2017, 2019)
Benzene	The average concentrations of benzene, expressed in micrograms per cubic meter of air ($\mu g/m^3$), in the period 2014–2016.	ISPRA (2015, 2016, 2017)

---

[1] The formula used was: $Excess_{mortality} = 100{,}000 * \left(\frac{deaths_{2020}}{pop_{2020}} - \frac{\overline{deaths}_{2015-2019}}{\overline{pop}_{2015-2019}}\right)$. Where $deaths_{2020}$ refers to the cumulative deaths from all causes registered from 1 March 2020 to 30 November 2020, $\overline{deaths}_{2015-2019}$ is the five-year average deaths (2015–2019) from all causes (from 1 March to 30 November), pop_{2020} means the population in the year 2020, and $\overline{pop}_{2015-2019}$ is the average population in the five-year period 2015–2019.

[2] The formula used was: $Pop_{density} = \frac{pop_{2020}}{square\_km}$. Where pop_{2020} means the population in the year 2020, and $square_{km}$ is the area of each province expressed in square kilometers.

[3] The pollution monitors are mainly located in the territory of the provincial's capital. This is true for all the air pollutants considered in this study.

BaP	The average concentrations of benzo[a]pyrene, expressed in expressed in nanogram per cubic meter of air (ng/m^3), in the period 2014–2018.	Istat (2015, 2017, 2019)
As	The average concentrations of arsenic, expressed in nanogram per cubic meter of air (ng/m^3), in the period 2014–2016.	ISPRA (2015, 2016, 2017)
Cd	The average concentrations of cadmium, expressed in nanogram per cubic meter of air (ng/m^3), in the period 2014–2016.	ISPRA (2015, 2016, 2017)
Ni	The average concentrations of nickel, expressed in nanogram per cubic meter of air (ng/m^3), in the period 2014–2016.	ISPRA (2015, 2016, 2017)

Bibligraphy (Table B1)

Associazione Italiana Gestori Aeroporti (ASSOAEROPORTI) (2021). https://assaeroporti.com/statistiche/. (Accessed 13 January 2021).

Eurostat (2013). Rural Development. “Overview Regions & cities”. https://ec.europa.eu/eurostat/web/rural-development/methodology. (Accessed 3 May 2021).

Istituto Superiore di Sanità (ISS) (2021a). Sorveglianza PASSI–EpiCentro–ISS. (Accessed 10 April 2021. https://www.epicentro.iss.it/passi/dati/sovrappeso

Istituto Superiore di Sanità (ISS) (2021b). Sorveglianza PASSI–EpiCentro–ISS. (Accessed 10 April 2021). https://www.epicentro.iss.it/passi/dati/fumo

Italian Institute for Environmental Protection and Research (ISPRA) (2017). XIII Rapporto Qualità dell’ambiente urbano – Edizione 2017 (Chapter: Qualità dell’aria). https://www.isprambiente.gov.it/it/pubblicazioni/stato-dellambiente/xiii-rapporto-qualita-dell2019ambiente-urbano-edizione-2017. (Accessed 25 March 2021).

Italian Institute for Environmental Protection and Research (ISPRA) (2016). XII Rapporto Qualità dell’ambiente urbano – Edizione 2016 (Chapter: Qualità dell’aria). https://www.isprambiente.gov.it/it/pubblicazioni/stato-dellambiente/xii-rapporto-qualita-dell2019ambiente-urbano-edizione-2016. (Accesed 25 March 2021).

Italian Institute for Environmental Protection and Research (ISPRA) (2015). Qualità dell’ambiente urbano – XI Rapporto. Edizione 2015 (Chapter: Qualità dell’aria). https://www.isprambiente.gov.it/it/pubblicazioni/stato-dellambiente/qualita-dellambiente-urbano-xi-rapporto.-edizione-2015. (Accessed 25 March 2021).

Italian Ministry of Health (2020). COVID-19, Dati Province. https://github.com/pcm-dpc/COVID-19/tree/master/dati-province. (Accessed 5 April 2021).

Italian National Institute of Statistics (ISTAT) (2015). Qualità dell’ambiente urbano – Fattori di pressione. https://www.istat.it/it/archivio/173187. (Accessed 15 April 2021).

Italian National Institute of Statistics (ISTAT) (2017). Ambiente Urbano. https://www.istat.it/it/archivio/207482. (Accessed 20 April 2021).

Italian National Institute of Statistics (ISTAT) (2019). Ambiente Urbano. https://www.istat.it/it/archivio/236912. (Accessed 22 April 2021).

Italian National Institute of Statistics (ISTAT) (2020). Temperatura e precipitazioni nelle città capoluogo di provincia. https://www.istat.it/it/archivio/242010. (Accessed 5 May 2021).

Ministero delle politiche agricole alimentari e forestali (Mipaaf) (2021). Osservatorio agroclimatico: statistiche provinciali. https://www.politicheagricole.it/flex/FixedPages/Common/miepfy700_provincie.php/L/IT (Accessed 20 May 2021).

Italian National Institute of Statistics (ISTAT) (2021a). I.Stat database. http://dati.istat.it (Accessed 12 May 2021 and 25 April 2022).

Italian National Institute of Statistics (ISTAT) (2021b). Principali Statistiche Geografiche sui Comuni. https://www.istat.it/it/archivio/156224. (Accessed 20 July 2021).

Italian National Institute of Statistics (ISTAT) (2022). Decessi e cause di morte: cosa produce l’ISTAT. https://www.istat.it/it/archivio/240401. (Accessed 22 April 2022).

Legambiente (2020). Ecosistema Urbano. Rapporto sulle performance ambientali delle città 2020. https://www.legambiente.it/wp-content/uploads/2020/11/Ecosistema-Urbano-2020.pdf. (Accessed 5 June 2021).

APPENDIX C1

Table C1. Summary descriptive statistics of the variables used in the empirical analysis.

Variables	N	Mean	S. D.	Minimum	Maximum
Cumulative case	107	14,704.92	20,977	945	152,125
Prevalence (1)	107	2.4478	1.0921	0.5266	5.141
Prevalence (2)	107	4.5985	1.6367	1.4627	10.0799
Excess deaths	107	854.35	1,316.47	44	9,463
Excess mortality (1)	107	157.88	118.46	25.15	624.77
Excess mortality (2)	107	209.89	122.48	37.0811	609.11
AUT border	107	0.028	0.1659	0	1
FRA border	107	0.0374	0.1906	0	1
SLO border	107	0.028	0.1659	0	1
SWI border	107	0.0561	0.2312	0	1
Aged 0-19	107	17.37	1.5277	14.142	21.323
Airport dist.	107	57.202	38.59	4	157
Foreigners	107	7.9627	3.3778	2.0113	18.4854
Male	107	48.806	0.4649	47.647	49.845
Pop. Density	107	269.81	382.25	36.99	2,616.7
Urbanization	107	1.8224	0.7113	1	3
Obesity	107	10.33	2.037	7.3	14.3
Smokers	107	24.75	1.9921	21.3	28.8
LRT disease	107	0.4085	0.0829	0.233	0.683
Large firms	107	4.8662	3.6827	0	21.7472
Capitals	107	0.1869	0.3917	0	1
Size of province	107	2,823.07	1,717.98	212.5	7,691.75
Altitude	107	239.99	244.72	0.9063	1,168.2
Rainy days	107	87.1	12.312	60.3	120.55
Temperature	107	13.197	2.7377	3.95	17.8
NO_2 (μg/m^3)	107	26.3279	9.2962	4	49.98
O_3 (>120 μg/m^3)	98	28.2256	21.0366	0	75.0778
O_3 (>180 μg/m^3)	95	8.9064	18.325	0	100.2
$PM_{2.5}$ (μg/m^3)	97	15.3609	5.6004	0	27
PM_{10} (μg/m^3)	107	24.9518	5.7673	15.1667	36.2083
PM_{10} (>50 μg/m^3)	107	25.1509	22.7931	0	81.58
Benzene (μg/m^3)	88	1.2069	0.5249	0.2	3.05
BaP (ng/m^3)	73	0.4363	0.3776	0	1.64
As (ng/m^3)	60	0.9559	0.7165	0.2	3.7
Cd (ng/m^3)	60	0.343	0.4056	0	2.4778
Ni (ng/m^3)	60	3.6301	2.5968	1.1333	16.2889

Notes: N, number of observations; S.D., standard deviation; abs., absolute. Prevalence (1) and prevalence (2) refer to the fraction of population infected on 30 November 2020, and on 28 February 2021, respectively. Excess mortality (1) and excess mortality (2) refer to values registered on 30 November 2020, and on 28 February 2021, respectively.

APPENDIX D

Table D1. Cameron and Trivedi's (1990) decomposition of IM-test (residuals from OLS models reported in table 9)

Prevalence	Heteroscedasticity	Skewness	Kurtosis
Model 1	107	24.78	2.31
	[0.4545]	[0.21]	[0.1287]
Model 2	107	22.74	0.01
	0.4545	[0.302]	[0.9318]
Model 3	98	23.77	2.04
	[0.4525]	[0.2526]	[0.1529]
Model 4	95	25.74	0.45
	[0.4517]	[0.1745]	[0.5007]
Model 5	97	28.21	1.29
	[0.4522]	[0.1045]	[0.2558]
Model 6	107	22.94	0.49
	[0.4545]	[0.2918]	[0.4828]
Model 7	107	24.72	0.81
	[0.4545]	[0.2122]	[0.3695]
Model 8	88	15.89	0.47
	[0.4498]	[0.7235]	[0.4941]
Model 9	73	14.37	0.4
	[0.4449]	[0.811]	[0.5256]
Model 10	60	20.92	0.89
	[0.4392]	[0.4016]	[0.3454]
Model 11	60	20.72	0.88
	[0.4392]	[0.4141]	[0.3478]
Model 12	60	20.94	0.16
	[0.4392]	[0.4008]	[0.6855]

Notes: p-value in parentheses.

Table D2. Cameron and Trivedi's (1990) decomposition of IM-test (residuals from OLS models reported in table 10)

Excess mortality	Heteroscedasticity	Skewness	Kurtosis
Model 1	107	22.32	1.69
	[0.4545]	[0.2685]	[0.1936]
Model 2	107	29.94	1.98
	0.4545	[0.0526]	[0.1591]
Model 3	98	21.37	1.89
	[0.4525]	[0.3169]	[0.1691]
Model 4	95	17.56	1.04
	[0.4517]	[0.5522]	[0.3069]
Model 5	97	35.62	2.45
	[0.4522]	[0.0118]	[0.1174]
Model 6	107	26.02	3.22
	[0.4545]	[0.1297]	[0.0728]
Model 7	107	26.5	3.19
	[0.4545]	[0.1169]	[0.0741]
Model 8	88	27.07	1.94
	[0.4498]	[0.103]	[0.1639]
Model 9	73	22.52	1.48
	[0.4449]	[0.2589]	[0.2231]
Model 10	60	22.69	1.71

	[0.4392]	[0.2512]	[0.1913]
Model 11	60	20.55	1.17
	[0.4392]	[0.3619]	[0.2784]
Model 12	60	20.68	1.16
	[0.4392]	[0.3546]	[0.2814]

Notes: p-value in parentheses. P-value is underlined when it is less than 0.05.

Figure D3. Histogram of residuals (Model 5, Table D2).

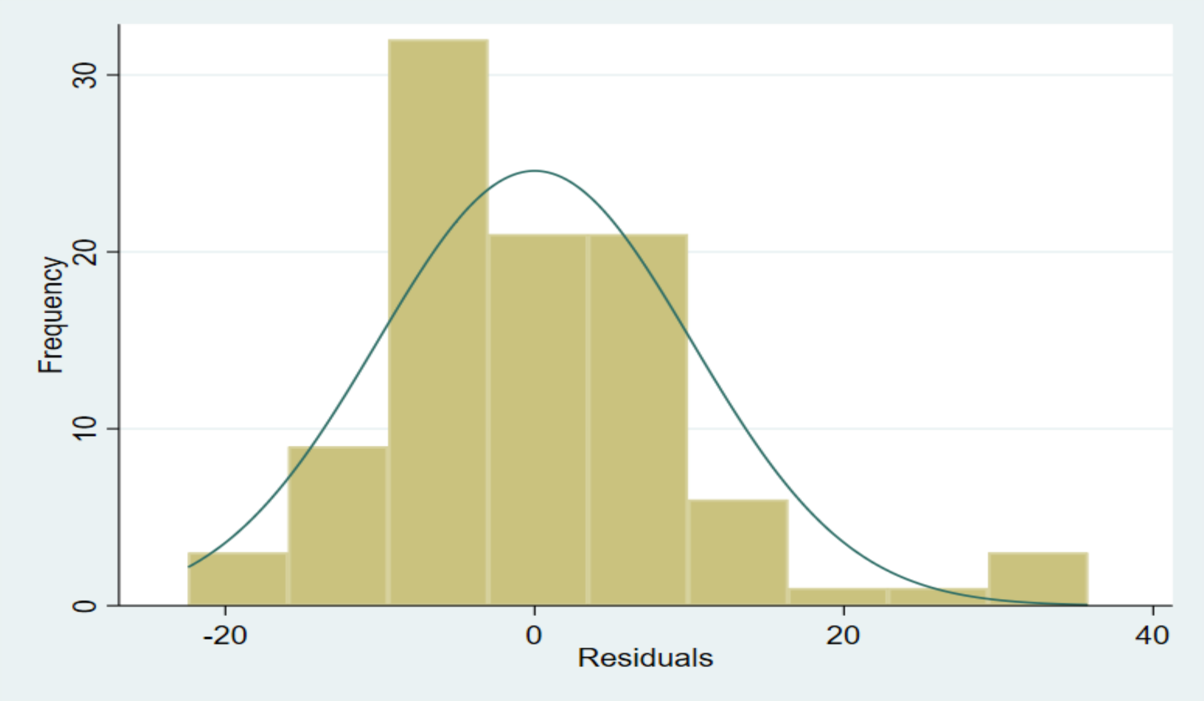

APPENDIX E

Table E1. Results of Moran's I for main variables.

Variables	I	E(I)	Sd(I)	Z	p-value
Prevalence (1)	0.341	-0.009	0.014	24.648	0.000
Prevalence (2)	0.265	-0.009	0.014	19.398	0.000
Excess mort. (1)	0.362	-0.009	0.014	26.476	0.000
Excess mort. (2)	0.363	-0.009	0.014	26.33	0.000
Aged 0-19	0.09	-0.009	0.014	6.971	0.000
Foreigners	0.316	-0.009	0.014	22.918	0.000
Male	0.051	-0.009	0.014	4.282	0.000
Pop. Density	0.045	-0.009	0.013	4.229	0.000
Urbanization	0.068	-0.009	0.014	5.47	0.000
Obesity	0.295	-0.009	0.014	21.382	0.000
Smokers	0.154	-0.009	0.014	11.475	0.000
LRT disease	0.08	-0.009	0.014	6.355	0.000
Large firms	0.24	-0.009	0.014	17.77	0.000
Altitude	0.032	-0.009	0.014	2.951	0.002
Rainy days	0.085	-0.009	0.014	6.661	0.000
Temperature	0.228	-0.009	0.014	16.911	0.000

Notes: I, Global Moran's I; E(I); expected index value; Sd, standard deviation; Z, z-score. Prevalence (1) and (2) refer to 30 November 2020 and 28 February 2021, respectively. The same applies for excess mort. (1) and (2).
